# Supplementary material for: DNA framework-engineered chimeras platform enables selectively targeted protein degradation
Source: Nat Commun. 2023 Jul 27;14:4510. doi: 10.1038/s41467-023-40244-7 (PMC10372072; doi:10.1038/s41467-023-40244-7)
Supplement: Supplementary file 1 — Supplementary Information [file 41467_2023_40244_MOESM1_ESM.pdf]

Supplementary information for

DNA framework-engineered chimeras platform enables selectively targeted protein degradation

Li Zhou[1], Bin Yu[2], Mengqiu Gao[1], Rui Chen[2], Zhiyu Li[2,3], Yueqing Gu[1]*, Jinlei Bian[2,3]* and Yi Ma[1]*

* Yi Ma, yima@cpu.edu.cn; Jinlei Bian, bianjl@cpu.edu.cn; Yueqing Gu, guengineering@cpu.edu.cn

[1]Department of Biomedical Engineering, School of Engineering, China Pharmaceutical University, Nanjing 210009, China
[2]State Key Laboratory of Natural Medicines, China Pharmaceutical University, Nanjing 210009, China
[3]Jiangsu Key Laboratory of Drug Design and Optimization, Department of Medicinal Chemistry, China Pharmaceutical University, Nanjing 210009, China

This PDF file includes:

Supplementary Figures and Tables

Supplementary Note - Chemistry Synthesis

^{1}H NMR Spectra

^{13}C NMR Spectra

HNMS Spectra

HPLC Spectra

Supplementary Figures

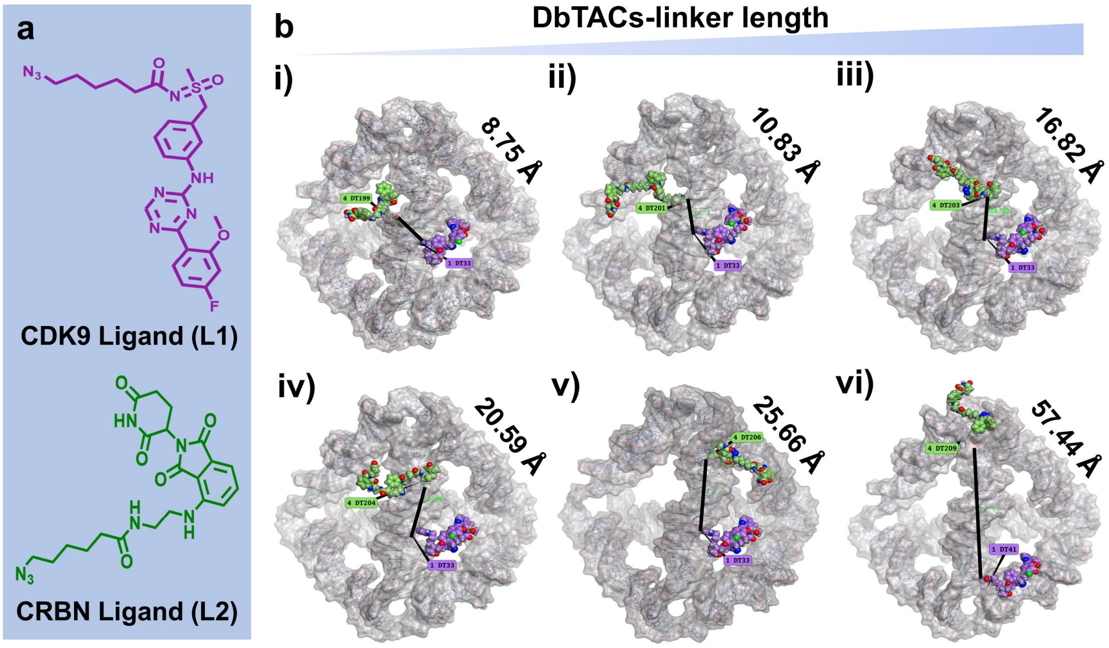

Supplementary Fig. 1. All-atom models of designed DbTACs generated by the PolygenDNA tool. (**a**) Chemical structures of the ligand molecules. (**b**) All-atom images of DbTACs with linker lengths of (i) 8 Å, (ii) 11 Å, (iii) 16 Å, (iv) 21 Å, (v) 26 Å, and (vi) 57 Å. Theoretical linker lengths were measured using MOE software. Purple, green, and gray indicate CDK9 ligand, CRBN ligand, and DNA tetrahedra, respectively.

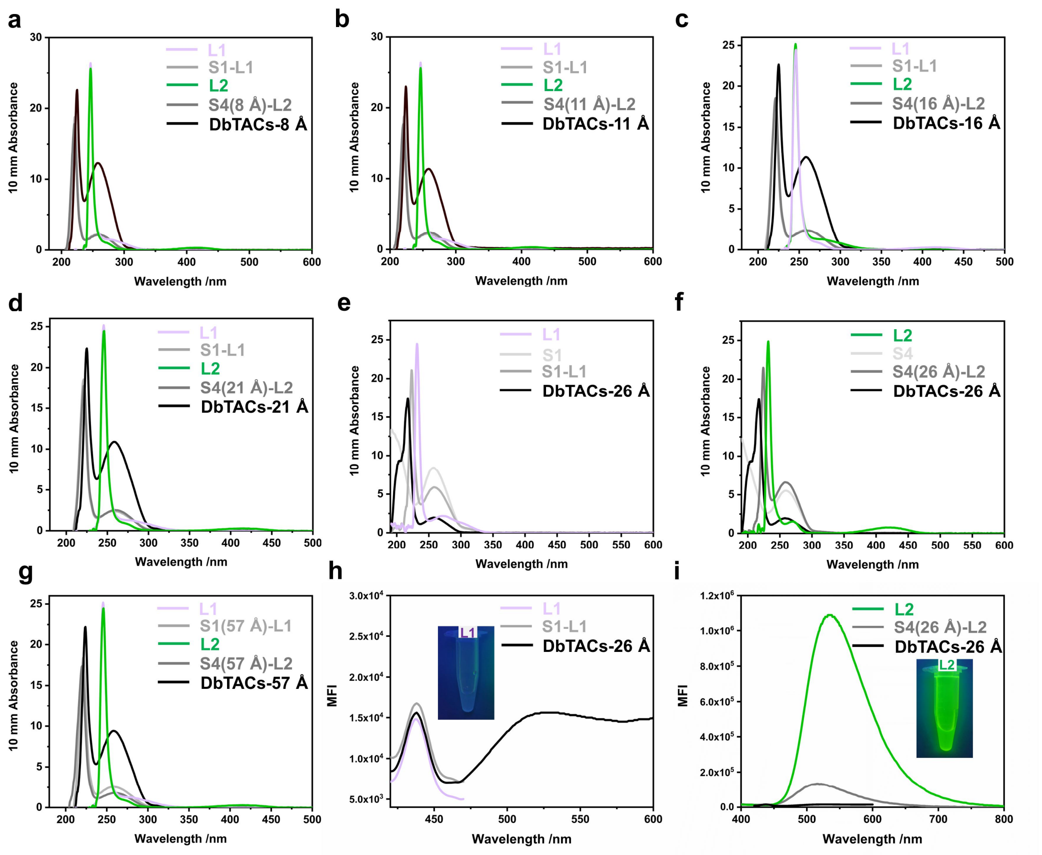

Supplementary Fig. 2. Spectroscopy characterization of DbTACs. UV–visible spectroscopy of (**a**) DbTACs-8 Å, (**b**) DbTACs-11 Å, (**c**) DbTACs-16 Å, (**d**) DbTACs-21 Å, (**e**) DbTACs-26 Å (L1, CDK9), (**f**) DbTACs-26 Å (L2, CRBN), and (**g**) DbTACs-57 Å. Fluorescence spectroscopy of (**h**) DbTACs-26 Å (L1, CDK9) and (**i**) DbTACs-26 Å (L2, CRBN).

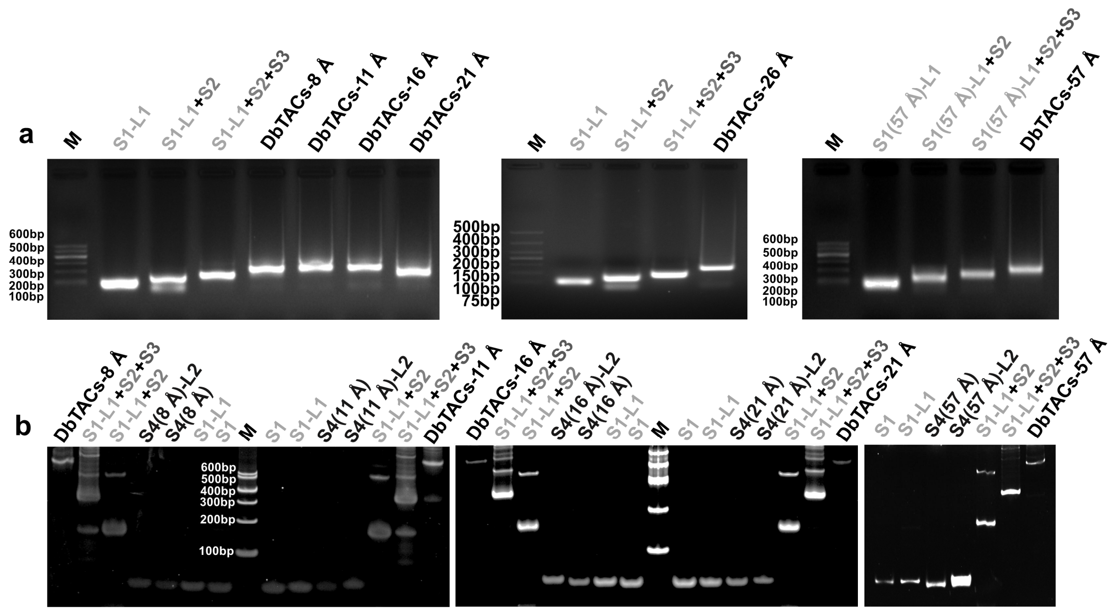

Supplementary Fig. 3. Gel electrophoresis of DbTACs. (**a**) Agarose gel electrophoresis of DbTACs-8 Å, -11 Å, -16 Å, -21 Å, -26 Å, and -57 Å self-assembly. (**b**) PAGE of covalent ligands and DbTACs-8 Å, -11 Å, -16 Å, -21 Å, and -57 Å self-assembly.

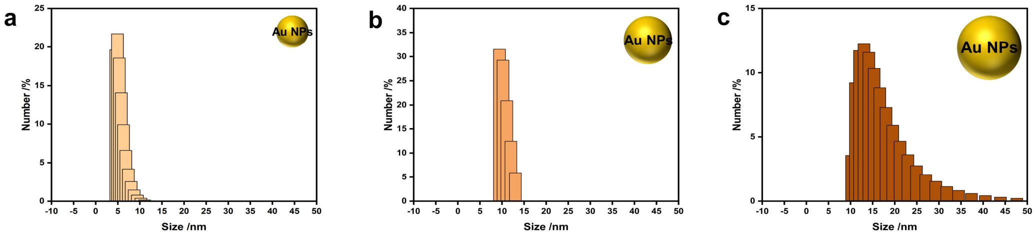

Supplementary Fig. 4. The hydrated particle size of Au NPs. The hydrated particle size distribution of (**a**) 5 nm, (**b**) 10 nm, and (**c**) 15 nm Au NPs were measured using a size analyzer.

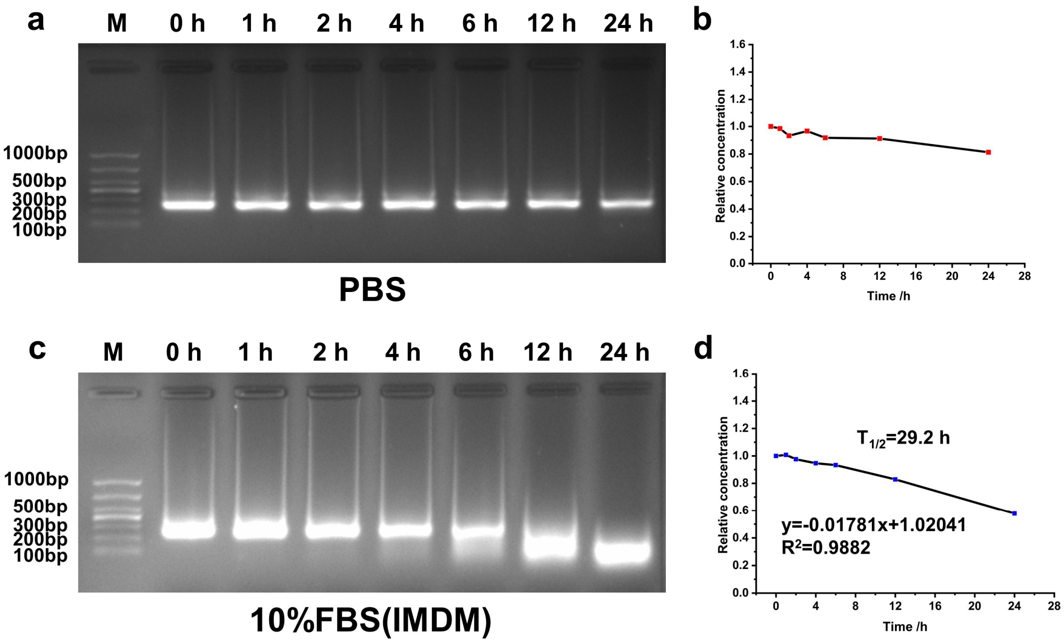

Supplementary Fig. 5. Stability analysis of DbTACs. (**a**) Stability analysis of DbTACs-26 Å in PBS at different time points (0, 1, 2, 4, 6, 12, and 24 h) by agarose gel electrophoresis and (**b**) the quantification of each bright band in (**a**). (**c**) Stability analysis of DbTACs-26 Å in 10% FBS (IMDM) at different time points (0, 1, 2, 4, 6, 12, and 24 h) by agarose gel electrophoresis and (**d**) the quantification of each bright band in (**c**), and non-linear fitting was performed to calculate $T_{1/2}$.

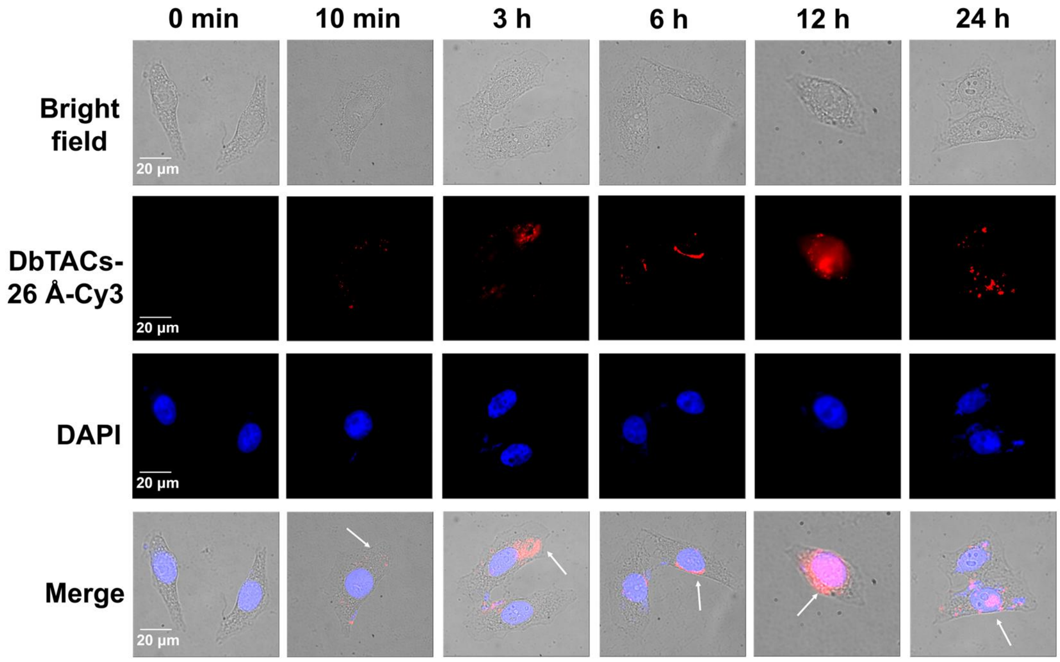

Supplementary Fig. 6. Subcellular localization of DbTACs-26 Å in HepG2 cells. HepG2 cells were treated with DbTACs-26 Å-Cy3 for different durations (0, 10 min, 3 h, 6 h, 12 h, and 24 h) and subsequently stained with DAPI. The red fluorescence represents the labeling of DbTACs-26 Å, while the blue color indicates the cell nucleus stained with DAPI. The Merge layer in the image displays the colocalization of DbTACs-26 Å, indicated by a white arrow. Scale bar: 20 μm.

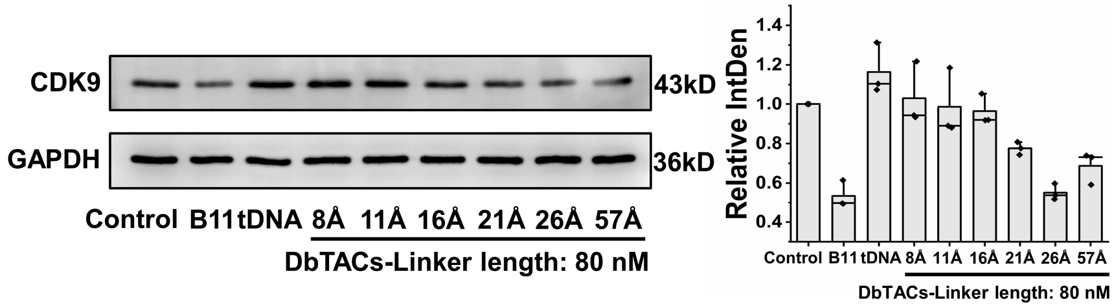

Supplementary Fig. 7. The degradation effect of DbTACs. WB assay of 80 nM DbTACs with different linker lengths for CDK9 degradation, and their semi-quantitative analysis. The error bars indicate the mean ± SD values; n=3.

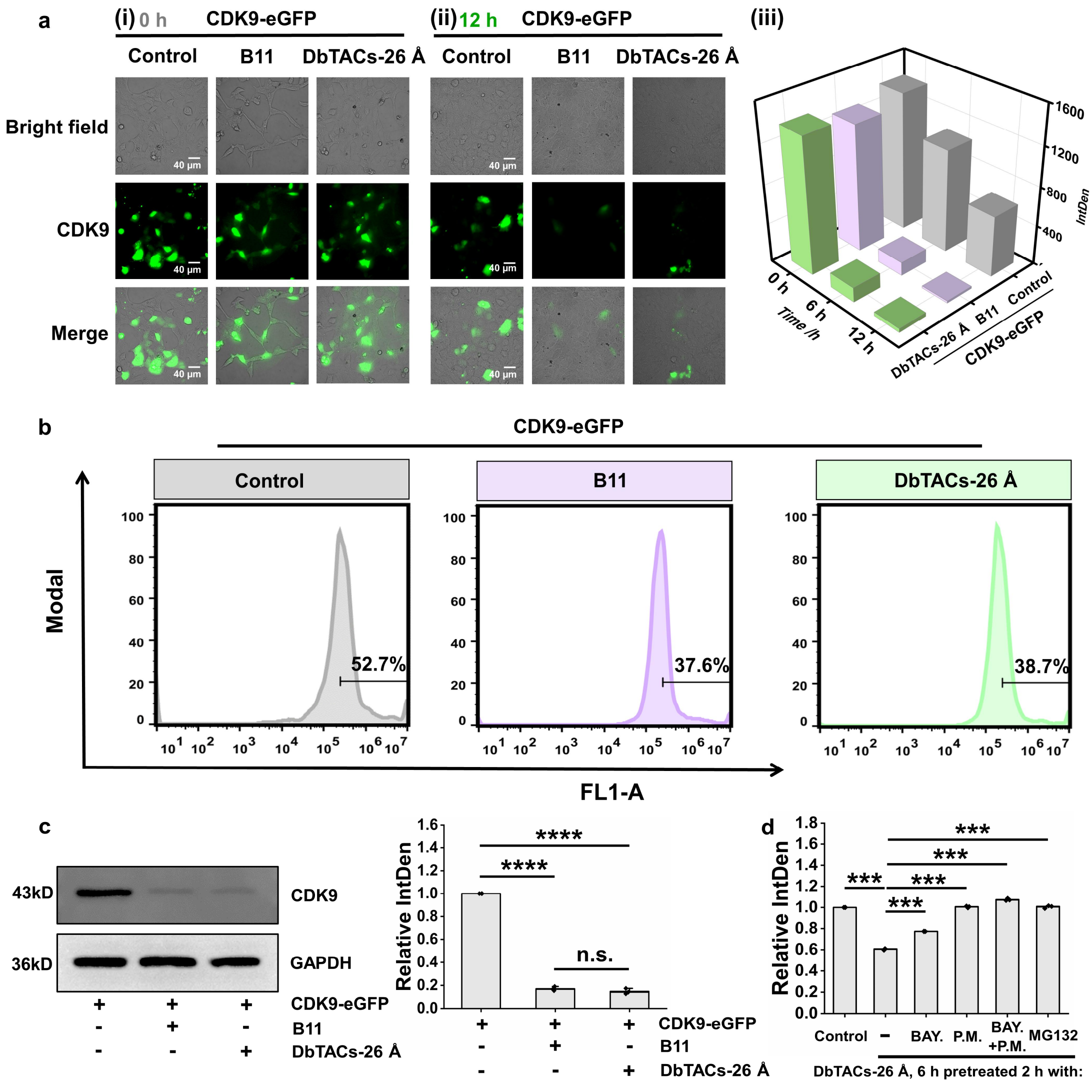

Supplementary Fig. 8. Real-time monition of CDK9 protein degradation in living cells. (**a**) Live-cell fluorescence microscopy imaging of HEK293T cells highly expressed CDK9-eGFP treated with DbTACs-26 Å (final concentration of 200 nM) for (i) 0 h and (ii)12 h. (iii) Semi-quantitative analysis of fluorescence intensity in HEK293T cells from different treatment groups at 0 h, 6 h, and 12 h. Scale bar = 40 μm. (**b**) Flow cytometry analysis of CDK9 content after plasmid-transfected HEK293T cells with high CDK9-eGFP expression were incubated with treatment groups for 12 h. The FL1-A channel represented eGFP. (**c**) Western blot assay of CDK9 content in HEK293T cells treated with control, B11 and DbTACs-26 Å for 12 h after plasmid transfection with fusion protein expressing CDK9-eGFP, along with quantitative analysis of gray value. Unpaired two-tailed t-test was used to evaluate statistical significance. Statistical significance was accepted with ****: P<0.0001 (CDK9-eGFP v.s. B11, CDK9-eGFP v.s. DbTACs-26 Å) and n.s.: no statistical difference. The error bars indicate the mean ± SD values; n = 3 biologically independent experiments. (**d**) Semi-quantitative analysis of ligand competition test. Paired two-tailed t-test was used to evaluate statistical significance. Statistical significance was accepted with ***: P=0.0001 (Control v.s. DbTACs-26 Å), P=0.0005 (BAY. v.s. DbTACs-26 Å), P=0.0005 (P.M. v.s. DbTACs-26 Å), P=0.0002 (BAY. + P.M. v.s. DbTACs-26 Å), and P=0.0005 (MG132 v.s. DbTACs-26 Å). The error bars indicate the mean ± SD values; n = 3 biologically independent experiments.

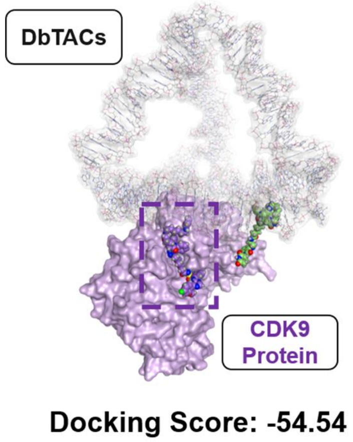

Supplementary Fig. 9. Molecular docking of DbTACs-26 Å. Molecular docking mapping of (A) DbTACs-26 Å docking with CDK9 protein.

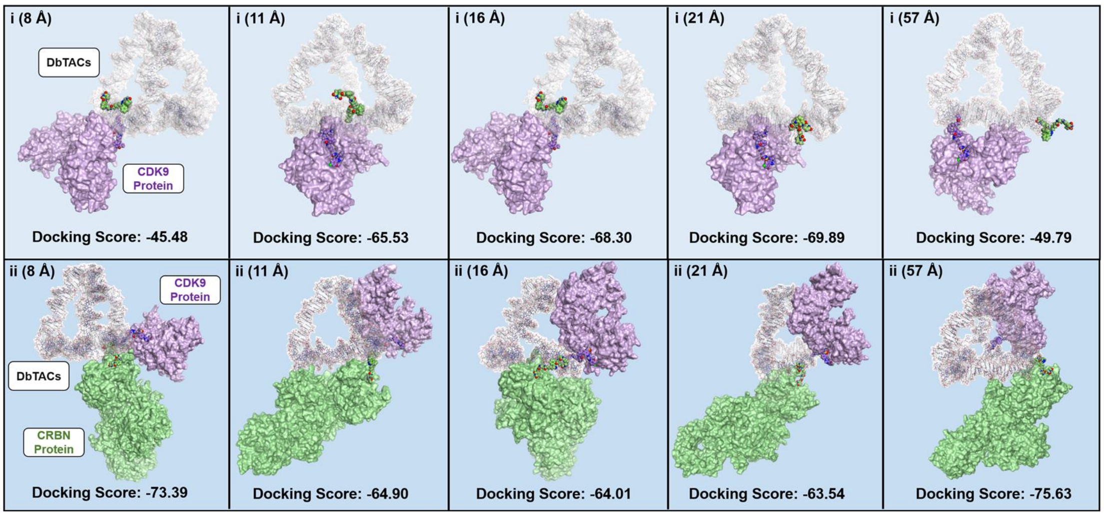

Supplementary Fig. 10. Molecular docking of DbTACs. The molecular docking images of (i) binary and (ii) ternary complex. Gray represents DbTACs, docked at the binding sites of the target protein. Green represents the CRBN E3 ligase and purple represents the CDK9 protein.

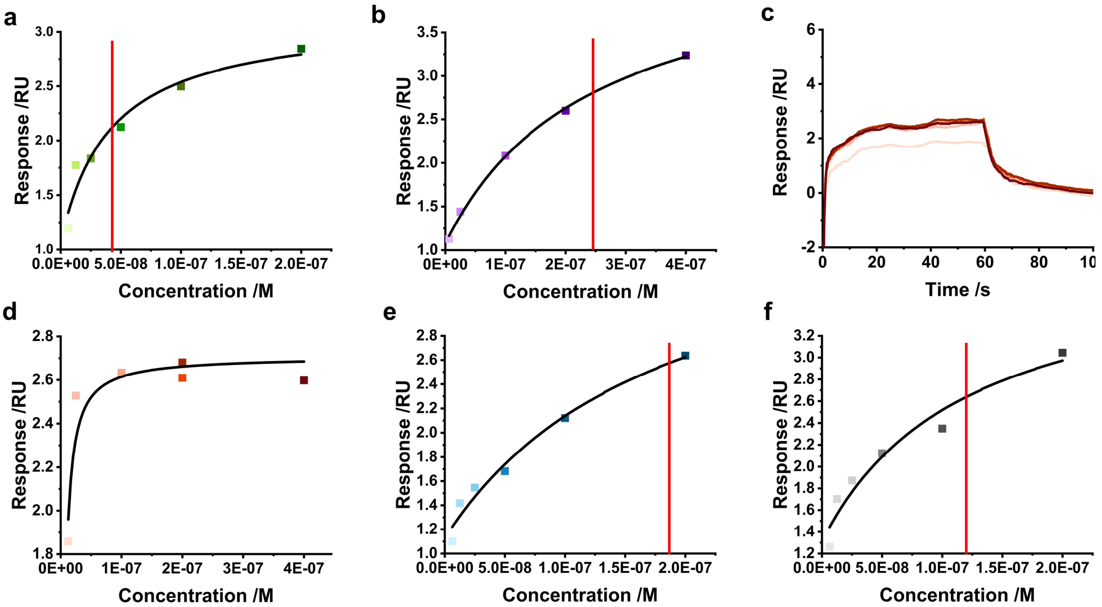

Supplementary Fig. 11. SPR analysis of complex formation with DbTACs. SPR sensorgrams and steady-state affinity (SSA) fitting for (**a**) DbTACs-26 Å+CRBN (ternary) or (**b**) DbTACs-26 Å (binary) binding to immobilized CDK9. (**c**) SPR kinetics and (**d**) SSA fitting of free CRBN protein binding to immobilized CDK9. SPR sensorgrams and SSA fitting for the ternary complex of (**e**) DbTACs-8 Å+CRBN or (**f**) DbTACs-57 Å+CRBN binding to immobilized CDK9.

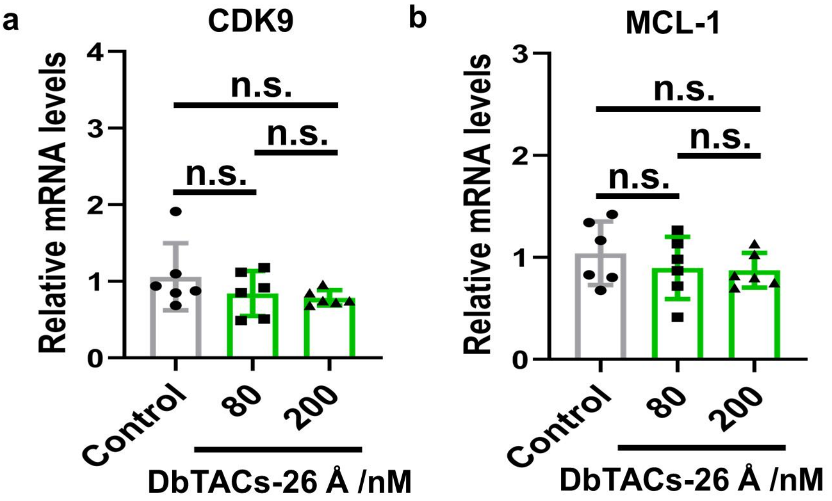

Supplementary Fig. 12. Validation of mRNA levels in MV4-11 cells treated with DbTACs. qRT–PCR verification of (**a**) *CDK9* and (**b**) *MCL-1* mRNA levels. The error bars indicate the mean ± SD values; n = 6. Unpaired two-tailed t-test was used to evaluate statistical significance. And n.s. represents for no significance.

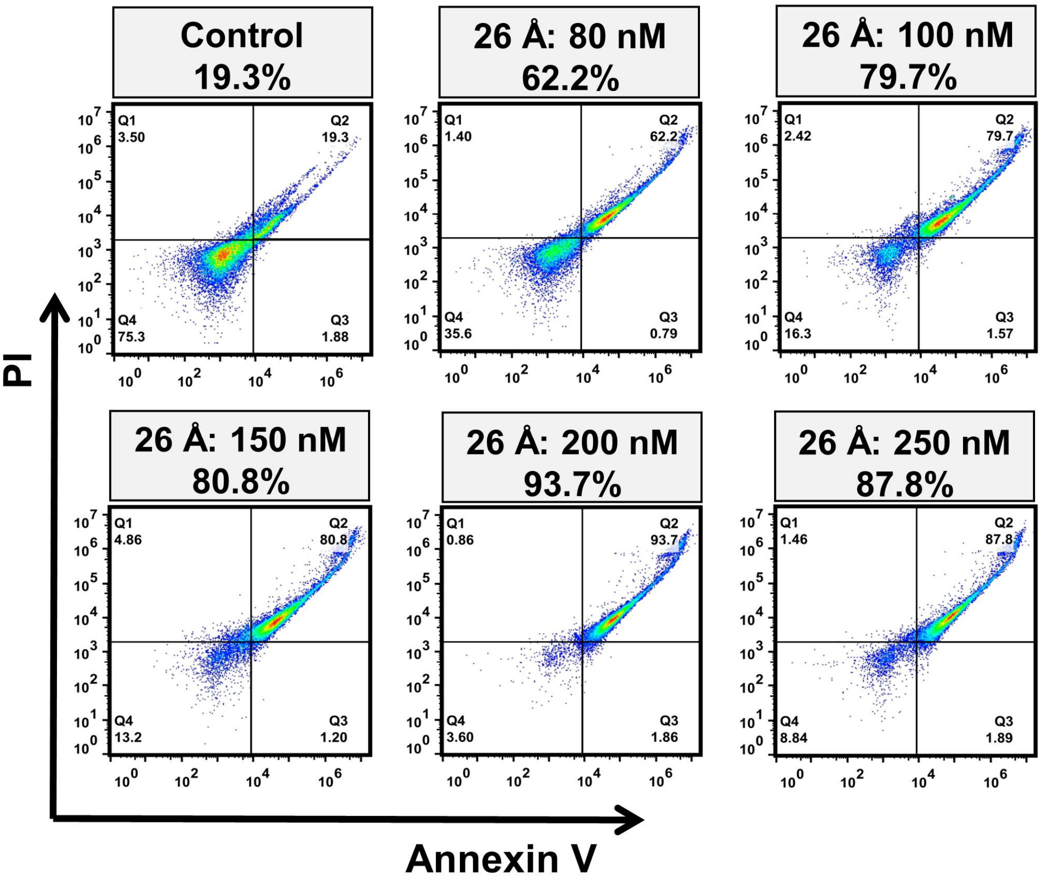

Supplementary Fig. 13. Cell apoptosis effect of DbTACs in MV4-11 cells. MV4-11 cells were treated with DbTACs-26 Å (80, 100, 150, 200, and 250 nM) for 6 h, then co-stained with annexin V/PI according to the instructions, and cell apoptosis was detected by flow cytometry. The annexin V/PI intensity dot plots showed the intensity of dot in the Q2 region increased significantly.

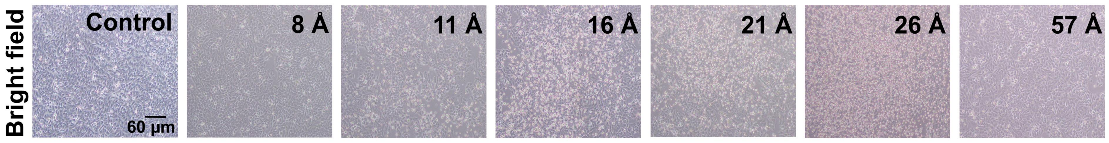

Supplementary Fig. 14. Cytotoxicity of DbTACs. HepG2 cells were inoculated in 6-well plates, and the growth states of cells in each group were photographed by a biological microscope after various DbTACs for 6 h were added. Scale bar = 60 μm.

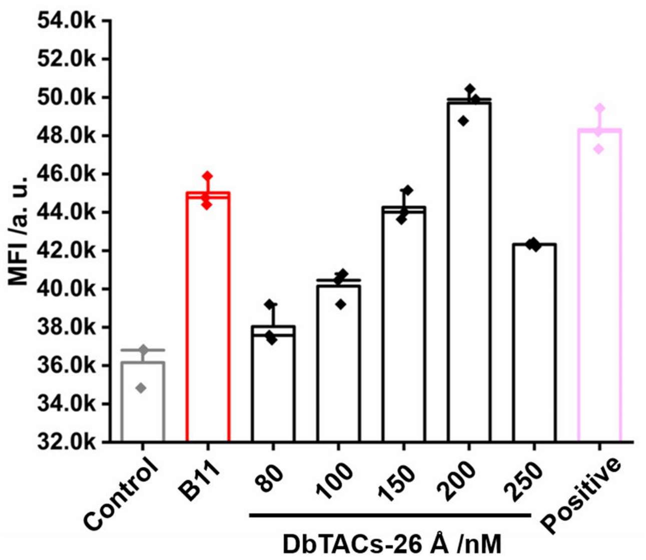

Supplementary Fig. 15. Activation of autophagy by DbTACs-26 Å treatment. Fluorescence microplate showing the levels of autophagy in MV4-11 cells following a 12-hour treatment with various compounds. An autophagy inducer (Earle's Balanced Salt Solution) was used as a positive control. OD values at excitation wavelength 335 nm and emission wavelength 512 nm were recorded. The error bars indicate the mean ± SD values; n = 3.

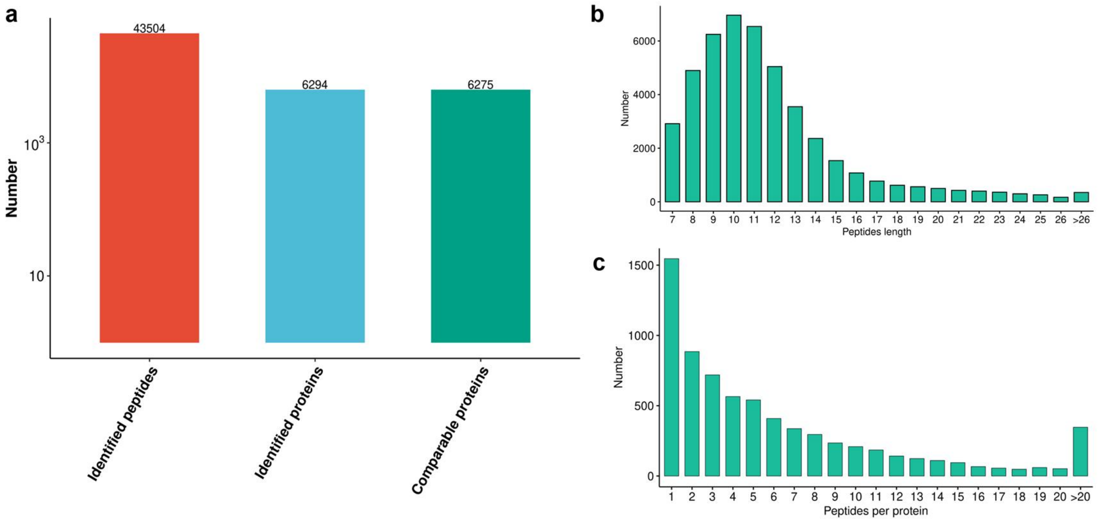

Supplementary Fig. 16. Quality check of proteomic data. a, The overview of all identified proteins. **b**, The distribution of peptides length among the identified peptides after trypsin digestion. **c**, The distribution of the number of peptides contained in the proteins.

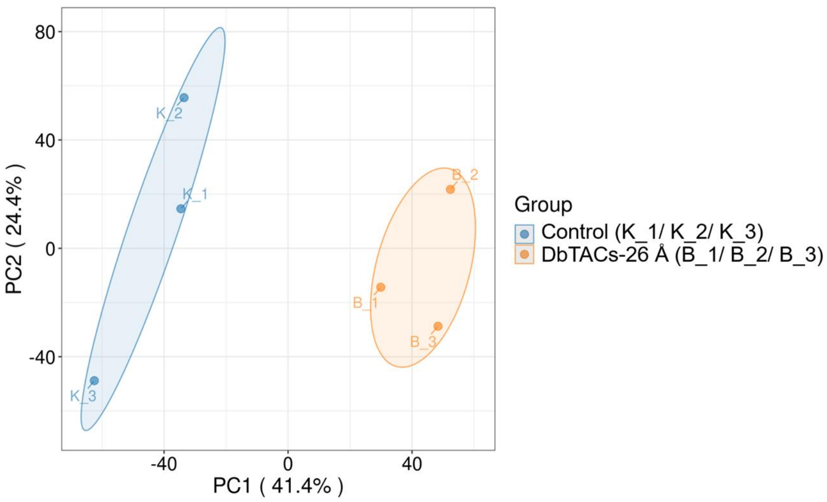

Supplementary Fig. 17. Principal component analysis (PCA) analysis. PCA analysis in MV4-11 cells treated with DbTACs-26 Å or control groups. The analysis was conducted using a dataset comprising replicates from three independent experiments (n=3).

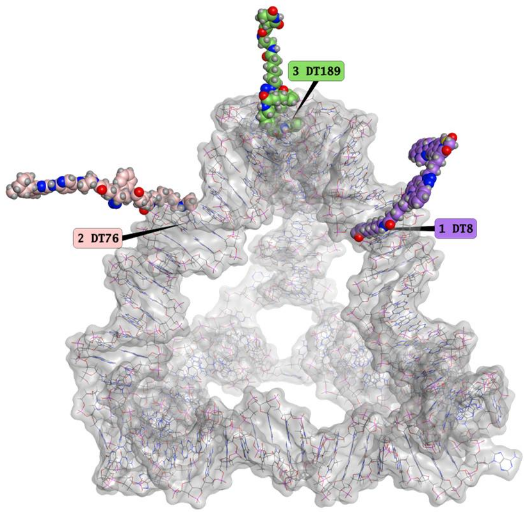

Supplementary Fig. 18. All-atom model diagram of bis-DbTACs. The all-atom structure of bis-DbTACs was drawn by polygenDNA and MOE tools. The lengths between CRBN ligand (green) and CDK9 ligand (purple) or CDK6 ligand (pink) are 26.88 Å and 29.12 Å, respectively.

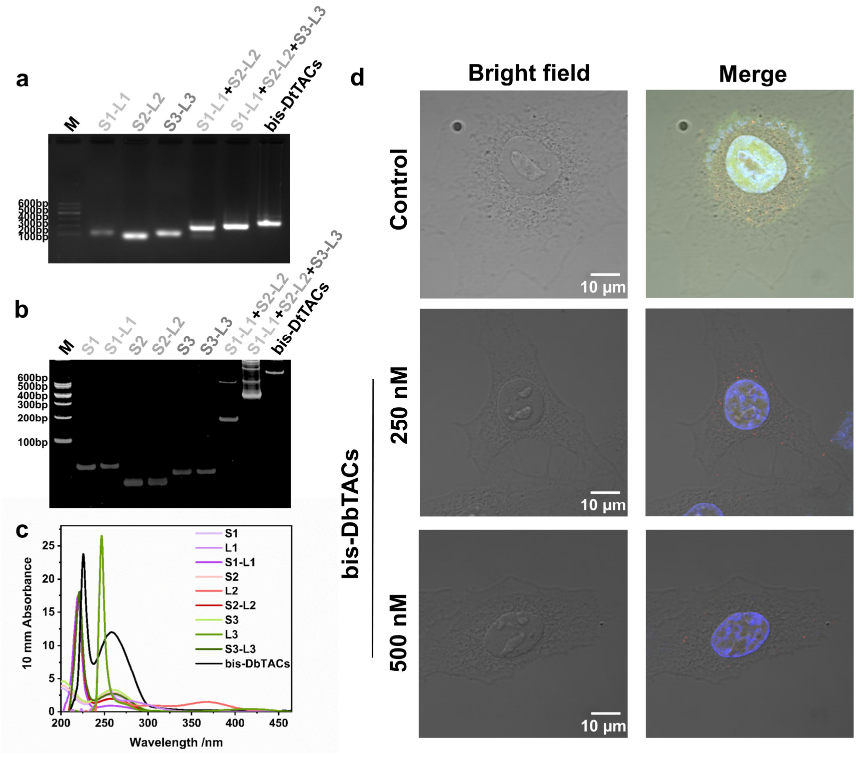

Supplementary Fig. 19. Characterization and effects of bis-DbTACs. (**a**) Agarose gel electrophoresis, (**b**) PAGE, and (**c**) UV–visible spectrum of ligands anchoring and self-assembly of bis-DbTACs. (**d**) Immunofluorescence double-staining images of HepG2 cells treated with/without bis-DbTACs for 6 h using laser scanning confocal microscope. Scale bar = 10 μm.

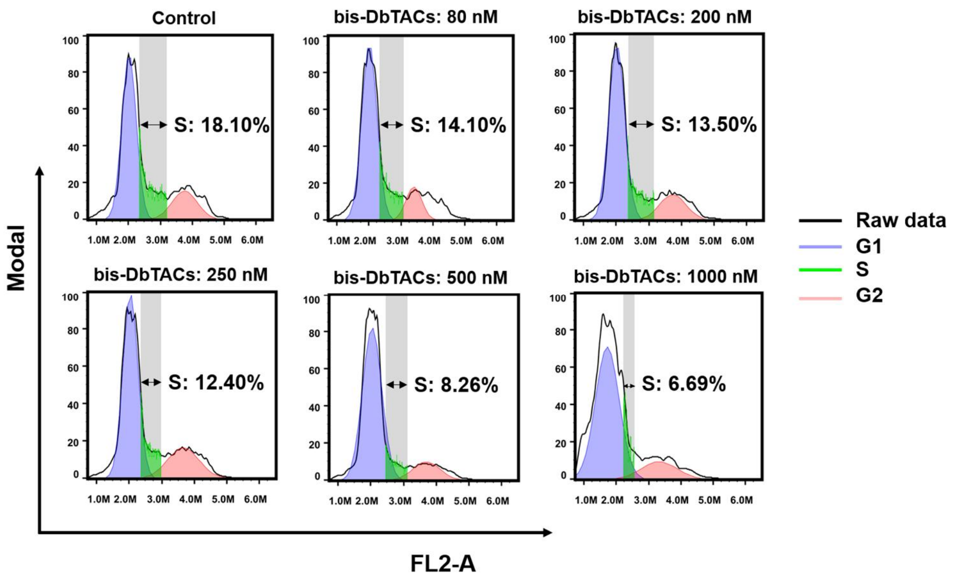

Supplementary Fig. 20. Cell cycle analysis of bis-DbTACs. After a 12-hour treatment with PBS (Control) or the specified concentration of bis-DbTACs, cells were collected and fixed overnight at 4°C and subjected to staining with PI/RNase A solution. Flow cytometric analysis was then performed to evaluate the cell cycle distribution. The reduction in the proportion of cells in the S phase indicated that bis-DbTACs led to impaired DNA synthesis. The FL2-A channel was used to measure PI fluorescence.

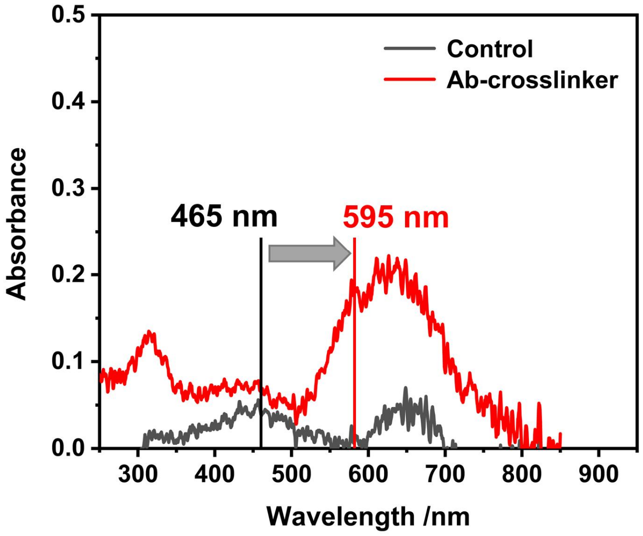

Supplementary Fig. 21. UV–visible spectroscopy of antibody-coupled crosslinker. The crosslinking between Ab and crosslinker BMPS was verified by Bradford assay.

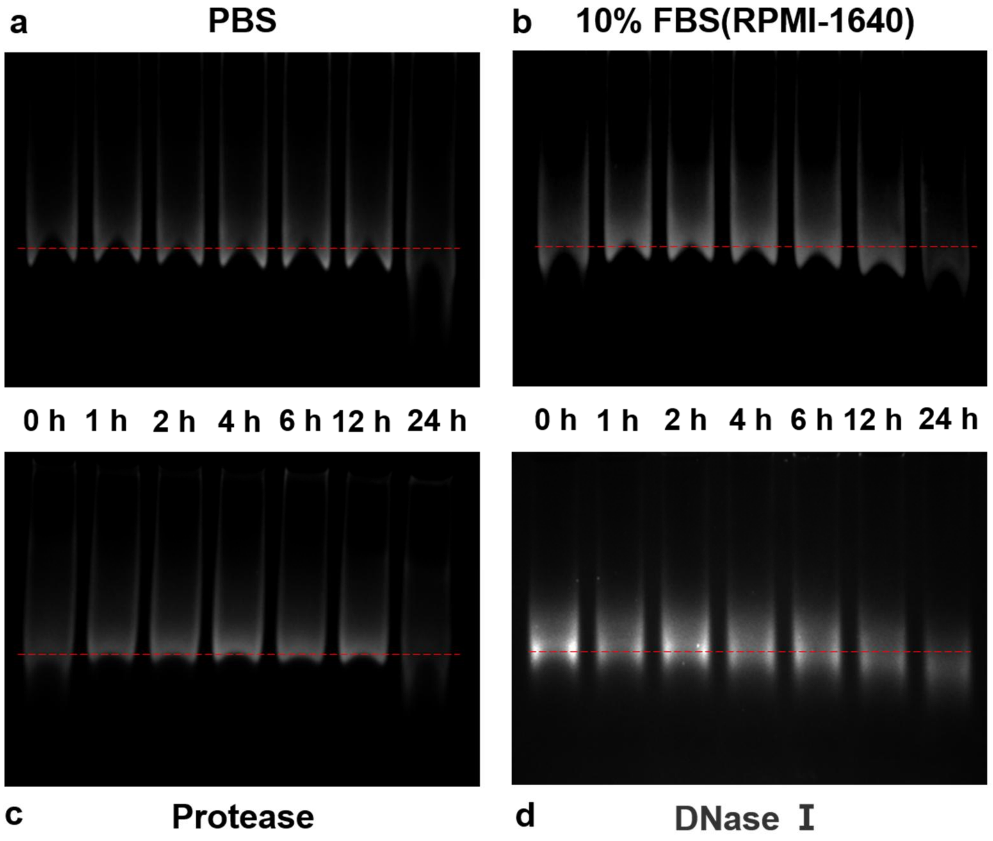

Supplementary Fig. 22. Stability analysis of Abs-DbTACs. Stability analysis of Abs-DbTACs in (**a**) PBS, (**b**) 10%FBS (RPMI-1640), (**c**) Protease, and (**d**) DNase I at different time points (0, 1, 2, 4, 6, 12, and 24 h) by agarose gel electrophoresis. The red dotted lines indicate the same horizontal position of the gels.

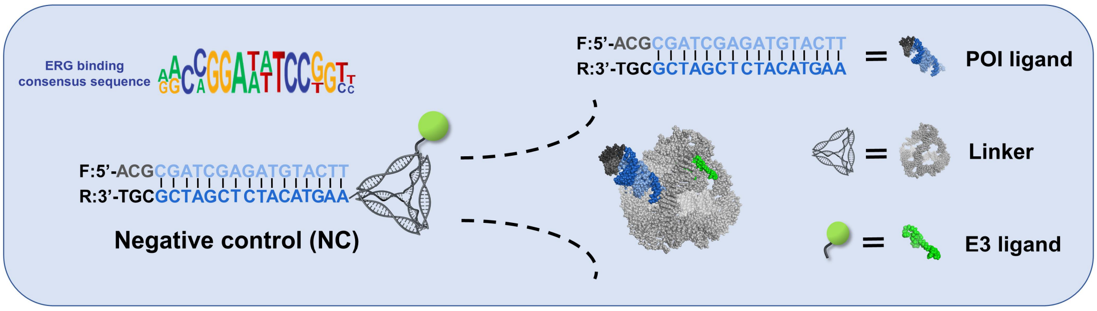

Supplementary Fig. 23. Schematic diagram of negative control DbTACs formed by a non-specific sequence. A non-specific sequence (ACGCGATCGAGATGTACTT) was chosen as an isotype control based on the in silico prediction by the PROMO software (version 8.3 of TRANSFAC) DNA motif as POI ligand.

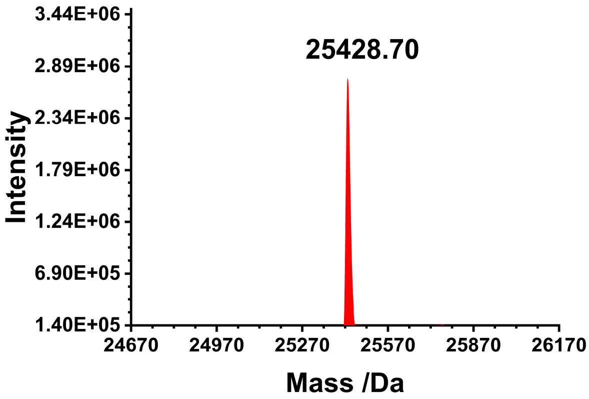

Supplementary Fig. 24. MS of DNA strand coupled reverse ERG binding consensus sequence. The theoretical and measured molecular weight were 25433.47 Da and 25428.70 Da, respectively. The acceptable molecular weight error is less than 5 ‰.

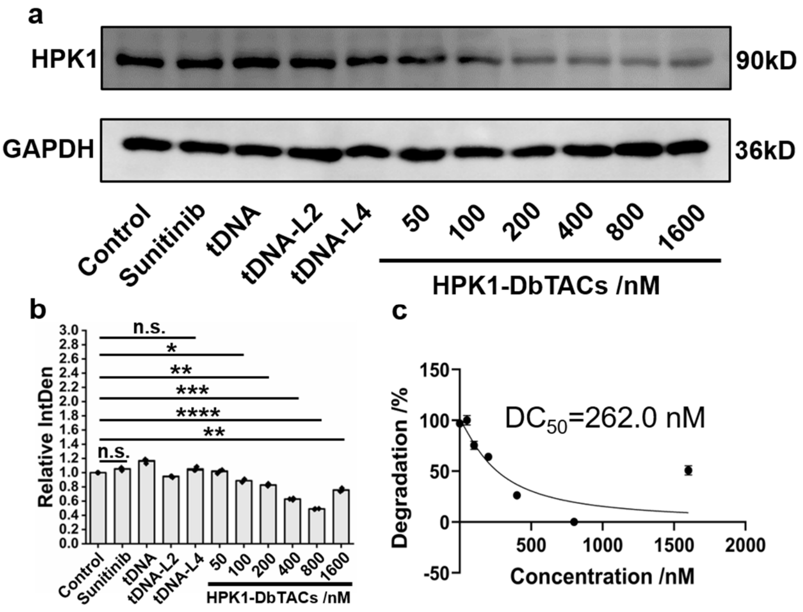

Supplementary Fig. 25. Degradation of cytoplasmic proteins by DbATCs. a) WB analysis of the degradation of cytoplasmic HPK1 target in Jurkat cells by HPK1-DbTACs (final concentrations of 50, 100, 200, 400, 800, and 1600 nM) and negative controls (Control, Sunitinib, tDNA, tDNA-L2, and tDNA-L4). GAPDH was used as a loading control. **b**) Semi-quantitative analysis of the intensity of the bands shown in (a). Paired two-tailed t-test was used to evaluate statistical significance. Statistical significance was indicated as follows: *: P=0.0117 (Control v.s. 100 nM HPK1-DbTACs), **: P=0.0030 (Control v.s. 200 nM HPK1-DbTACs) and P= 0.0034 (Control v.s. 1600 nM HPK1-DbTACs). ***: P= 0.0004 (Control v.s. 400 nM HPK1-DbTACs), ****: P<0.0001 (Control v.s. 800 nM HPK1-DbTACs), and n.s. indicates no significance. The error bars indicate the mean ± SD values; n = 3. **c**) Fitted dose-degradation curve of the relationship between drug concentration and degradation in Jurkat cells. The DC_{50} value, representing the concentration at which 50% of the cell is degraded, was determined to be 262.0 nM. The error bars indicate the mean ± SD values; n = 3.

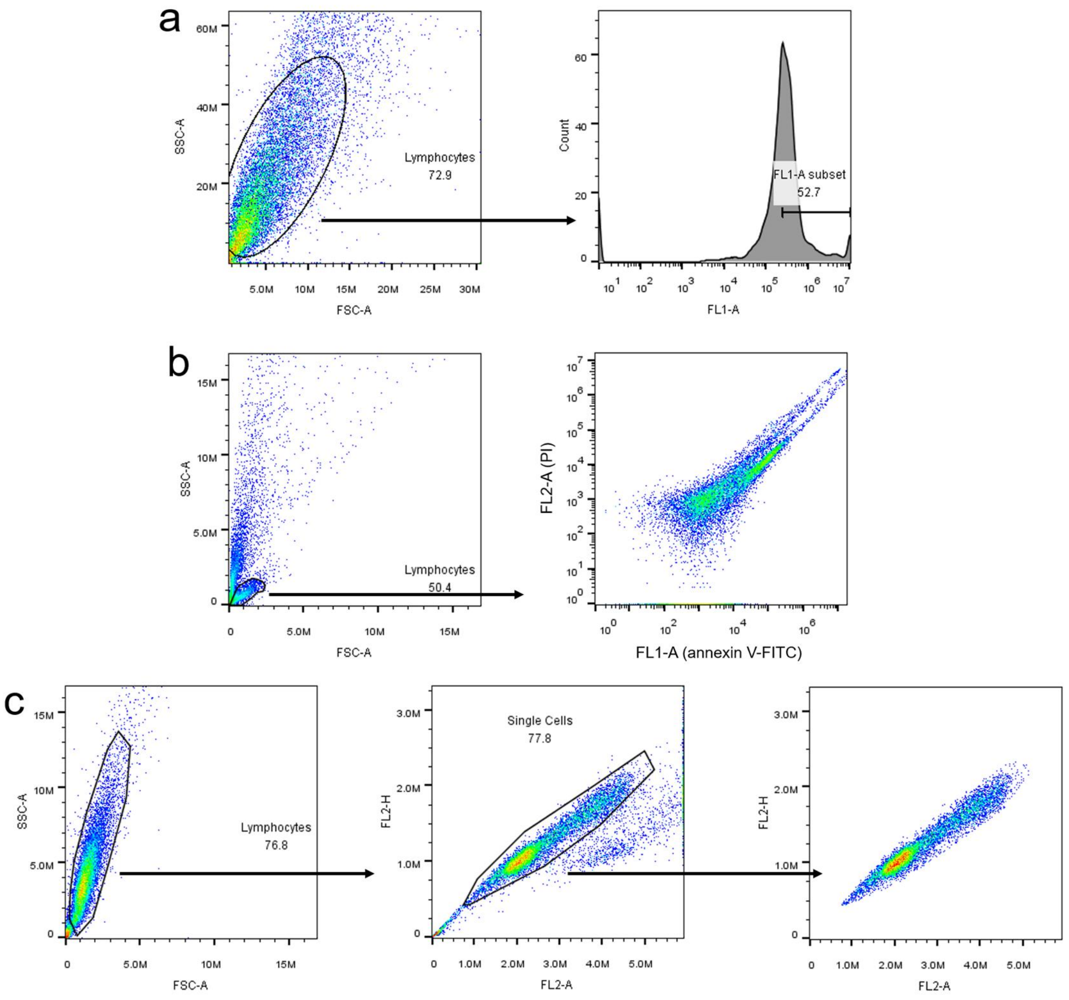

Supplementary Fig. 26. FACS gating strategies for (**a**) real-time monition of CDK9 protein degradation in plasmid-transfected HEK293T cells with high CDK9-eGFP expression (related to Supplementary Fig. 8b), (**b**) cell apoptosis (related to Supplementary Fig. 13) and (**c**) cell cycle (related to Supplementary Fig. 20).

Supplementary Tables

Supplementary Table 1. Sequences used in DbTACs-linker length (8 Å, 11 Å, 16 Å, 21 Å, 26 Å, 57 Å) and HPK1-DbTACs.

Name	Sequence (5'→3')
S1 (8 Å, 11 Å,16 Å, 21 Å, 26 Å)	CGGACTTGGGATTGCGTATGACTCAGAACAGA/iDBCOdT/[a]GTGATACGAGGATGGGCATGCTATGCCCGA
S1 (57 Å)	CGGACTTGGGATTGCGTATGAATCAGAACAGATGTGATAC/iDBCOdT/AGGATGGGCATGCTATGCCCGA
S2 (8 Å, 11 Å,16 Å, 21 Å, 26 Å, 57 Å)	CGTATGCAGTATTGCAATCGACGGGCATAGCATGCCCATCCACGGGCATAGCATGCACATGGA
S3 (8 Å, 11 Å,16 Å, 21 Å, 26 Å, 57 Å)	CATACGCAATCCCAAGTCCGACGATTGCAATACTGCATACGAGCTTGGTAAGCTTCAGACTTA
S4 (8 Å)	CGTATCACA/iDBCOdT/CTGTTCTGAGAAAGTCTGAAGCTTACCAAGCACCATGTGCATGCTATGCCCGA
S4 (11 Å)	CGTATCACATC/iDBCOdT/GTTCTGAGAAAGTCTGAAGCTTACCAAGCACCATGTGCATGCTATGCCCGA
S4 (16 Å)	CGTATCACATCTG/iDBCOdT/TCTGAGAAAGTCTGAAGCTTACCAAGCACCATGTGCATGCTATGCCCGA
S4 (21 Å)	CGTATCACATCTGT/iDBCOdT/CTGAGAAAGTCTGAAGCTTACCAAGCACCATGTGCATGCTATGCCCGA
S4 (26 Å)	CGTATCACATCTGTTC/iDBCOdT/GAGAAAGTCTGAAGCTTACCAAGCACCATGTGCATGCTATGCCCGA
S4 (57 Å)	AGTATCACATCTGTTCTGA/iDBCOdT/AAAGTCTGAAGCTTACCAAGCACCATGTGCATGCTATGCCCGA
S1 (HPK1-DbTACs)	CGGACTTGGGATTGCGTATGACTCAGAACAGA/iDBCOdT/GTGATACGAGGATGGGCATGCTATGCCCGA
S2 (HPK1-DbTACs)	CGTATGCAGTATTGCAATCGACGGGCATAGCATGCCCATCCACGGGCATAGCATGCACATGGA
S3 (HPK1-DbTACs)	CATACGCAATCCCAAGTCCGACGATTGCAATACTGCATACGAGCTTGGTAAGCTTCAGACTTA
S4 (HPK1-DbTACs)	CGTATCACATCTGTTC/iDBCOdT/GAGAAAGTCTGAAGCTTACCAAGCACCATGTGCATGCTATGCCCGA

[a] Red represents the bases in the DNA sequence that are modified with the dibenzocyclooctyne (DBCO) group.

Supplementary Table 2. Sequences used in bis-DbTACs.

Name	Sequence (5'→3')
S1 (bis-DbTACs)	CGGACTT/iDBCOdT/GGATTGCGTATGACTCAGAACAGATGTGATACGAGGATGGGCATGCTATGCCCGT
S2 (bis-DbTACs)	CGTATGCAGTAT/iDBCOdT/GCAATCGTCGGGCATAGCATGCCCATCCACGGGCATAGCATGCACATGGA
S3 (bis-DbTACs)	CATACGCAATCCAAAGTCCG/iDBCOdT/CGATTGCAATACTGCATACGAGCTTGGTAAGCTTCAGACTTA
S4 (bis-DbTACs)	CGTATCACATCTGTTCTGAGAAAGTCTGAAGCTTACCAAGCACCATGTGCATGCTATGCCCGA

Supplementary Table 3. Sequences used in Au-DbTACs-linker length (8 Å, 11 Å, 16 Å, 21 Å, 26 Å, 57 Å) and Au-bis-DbTACs equivalents.

Name	Sequence (5’→3’)
10 nm Au-S1a (8 Å, 11 Å, 16 Å, 21 Å, 26 Å)	CGGACTTGGGATTGCGTATGACTCAGAACAGATAAAAAAA AAAAAAAAAAAAAAAAAAAAAAA[b]
10 nm Au-S1b (8 Å, 11 Å, 16 Å, 21 Å, 26 Å)	GTGATACGAGGATGGGCATGCTATGCCCGA
10 nm Au-S1a (57 Å)	CGGACTTGGGATTGCGTATGAATCAGAACAGATGTGATAC TAAAAAAAAAAAAAAAAAAAAAAAAAAAAAA
10 nm Au-S1a (57 Å)	CGGACTTGGGATTGCGTATGAATCAGAACAGATGTGATAC TAAAAAAAAAAAAAAAAAAAAAAAAAAAAAA
10 nm Au-S1b (57 Å)	AGGATGGGCATGCTATGCCCGA
S2 (8 Å, 11 Å,16 Å, 21 Å, 26 Å, 57 Å)	CGTATGCAGTATTGCAATCGACGGGCATAGCATGCCCATCC ACGGGCATAGCATGCACATGGA
S3 (8 Å, 11 Å,16 Å, 21 Å, 26 Å, 57 Å)	CATACGCAATCCCAAGTCCGACGATTGCAATACTGCATAC GAGCTTGGTAAGCTTCAGACTTA
5 nm Au-S4a (8 Å)	CGTATCACATAAAAAAAAAAAAAAA
5 nm Au-S4b (8 Å)	CTGTTCTGAGAAAGTCTGAAGCTTACCAAGCACCATGTGCA TGCTATGCCCGA
5 nm Au-S4a (11 Å)	CGTATCACATCTAAAAAAAAAAAAAAA
5 nm Au-S4b (11 Å)	GTTCTGAGAAAGTCTGAAGCTTACCAAGCACCATGTGCAT GCTATGCCCGA
5 nm Au-S4a (16 Å)	CGTATCACATCTGTAAAAAAAAAAAAAAA
5 nm Au-S4b (16 Å)	TCTGAGAAAGTCTGAAGCTTACCAAGCACCATGTGCATGCT ATGCCCGA
5 nm Au-S4a (21 Å)	CGTATCACATCTGTTAAAAAAAAAAAAAAA
5 nm Au-S4b (21 Å)	CTGAGAAAGTCTGAAGCTTACCAAGCACCATGTGCATGCT ATGCCCGA
5 nm Au-S4a (26 Å)	CGTATCACATCTGTTCTAAAAAAAAAAAAAAA
5 nm Au-S4b (26 Å)	GAGAAAGTCTGAAGCTTACCAAGCACCATGTGCATGCTAT GCCCGA
5 nm Au-S4a (57 Å)	AGTATCACATCTGTTCTGATAAAAAAAAAAAAAAA
5 nm Au-S4b (57 Å)	AAAGTCTGAAGCTTACCAAGCACCATGTGCATGCTATGCCC GA

10 nm Au-S1a (bis-DbTACs)	CGGACTTTAAAAAAAAAAAAAAAAAAAAAAAAAAAAAA
10 nm Au-S1b (bis-DbTACs)	GGATTGCGTATGACTCAGAACAGATGTGATACGAGGATGG GCATGCTATGCCCGT
15 nm Au-S2a (bis-DbTACs)	CGTATGCAGTATTAAAAAAAAAAAAAAAAAAAAAAAAAA AAAAAAAAAAAAAAAAAAA
15 nm Au-S2b (bis-DbTACs)	GCAATCGTCGGGCATAGCATGCCCATCCACGGGCATAGCA TGCACATGGA
5 nm Au-S3a (bis-DbTACs)	CATACGCAATCCAAAGTCCGTAAAAAAAAAAAAAAA
5 nm Au-S3b (bis-DbTACs)	CGATTGCAATACTGCATACGAGCTTGGTAAGCTTCAGACTT A

[b] Red represents the equivalent polyA domains of the specific positions of the ligands in DbTACs.

Supplementary Table 4. Sequences used in Abs-DbTACs and Oligo-DbTACs.

Name	Sequence (5’→3’)
S1 (Abs-DbTACs)	HS_[c]TGTGATACGAGGATGGGCATGCTATGCCCGACGGACTTGG GATTGCGTATGACTCAGAACAGA
S2 (Abs-DbTACs)	CGTATGCAGTATTGCAATCGACGGGCATAGCATGCCCATCCAC GGGCATAGCATGCACATGGA
S3 (Abs-DbTACs)	CATACGCAATCCCAAGTCCGACGATTGCAATACTGCATACGAG CTTGGTAAGCTTCAGACTTA
S4 (Abs-DbTACs)	CGTATCACATCTGTTC/iDBCOdT/GAGAAAGTCTGAAGCTTACC AAGCACCATGTGCATGCTATGCCCGA
S1 (Oligo-DbTACs)	GTGATACGAGGATGGGCATGCTATGCCCGACGGACTTGGGATT GCGTATGACTCAGAACAGATAACCGGATTTCCGGTCCGT[d]
ERG(F)	ACGGACCGGAAATCCGGTT[e]
S1-NC (Oligo-DbTACs)	GTGATACGAGGATGGGCATGCTATGCCCGACGGACTTGGGATT GCGTATGACTCAGAACAGATAAGTACATCTCGATCGCGT[f]
NC(F)	ACGCGATCGAGATGTACTT[g]
S2 (Oligo-DbTACs)	CGTATGCAGTATTGCAATCGACGGGCATAGCATGCCCATCCAC GGGCATAGCATGCACATGGA
S3 (Oligo-DbTACs)	CATACGCAATCCCAAGTCCGACGATTGCAATACTGCATACGAG CTTGGTAAGCTTCAGACTTA
S4 (Oligo-DbTACs)	CGTATCACATCTGTTC/iDBCOdT/GAGAAAGTCTGAAGCTTACC AAGCACCATGTGCATGCTATGCCCGA

[c] Red represents the bases in the DNA sequence that are modified with the sulfydryl (HS) group.

[d] Red represents the bases in the reverse strand that recognize the ERG protein.

[e] Red represents the bases in the forward strand that recognize the ERG protein.

[f] Red represents the bases in the reverse strand that can't recognize the ERG protein.

[g] Red represents the bases in the forward strand that can't recognize the ERG protein.

Supplementary Note - Chemistry Synthesis

General information

Palbociclib was purchased from energy-chemical (Shanghai, China), and MG132 was purchased from MedChemExpress (Shanghai, China). N-Succinimidyl 3-maleimidoproppionate (BMPS) and tris(2-carboxyethyl) phosphine hydrochloride (TCEP) were purchased from Adamas-beta (Shanghai, China). All chemical reagents were used without further purification. The azide ligands including cereblon (CRBN), CDK6, CDK9, pomalidomide (P.M.), and BAY-1143572 (BAY., 3) were synthesized and purified by us.

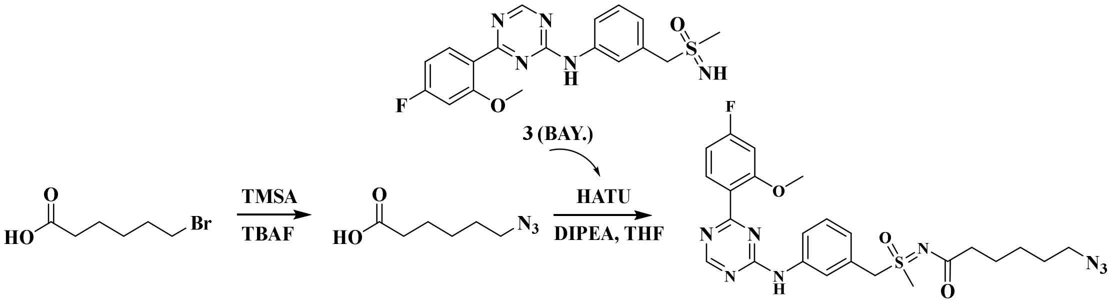

1 2 4 (CDK9 ligand, L1)

General procedure for the synthesis of compound 2. To a 25 mL three-neck round-bottom flask containing a magnetic stir bar was added 6-Bromohexanoic acid (0.200 g, 1.10 mmol), 5 mL of THF and trimethylsilylacetylene (TMSA, 0.154g, 1.34 mmol). The mixture was evacuated three times under an N_2 atmosphere. Then tetrabutylammonium fluoride (TBAF, 1.68 mL, 1.34 mmol) was added to the mixture and stirred at 65 °C for 3 h. Then the reaction solution was cooled to room temperature and extracted with EA three times. The organic layer was separated, concentrated to dryness, and used directly in the next step without purification. ^{1}H NMR (300 MHz, DMSO-d_6) δ 12.02 (s, 1 H), 3.35-3.30 (t, J = 7.5 Hz, 2 H), 2.25-2.20 (t, J = 7.5 Hz, 2 H), 1.59-1.48 (m, 4 H), 1.39-1.28 (m, 2 H). ^{13}C NMR (75 MHz, DMSO-d_6) δ 51.00, 33.97, 28.48, 26.20, 24.50. HR-MS (ESI) m/z: calculated for $C_6H_{11}N_3O_2$ $[M+H]^+$: 156.0779, found 156.1218.

General procedure for synthesis of compound 4 (CDK9 ligand, L1). The compound **2** (0.07 g, 0.464 mmol), DIPEA (0.20 g, 1.549 mmol) and HATU (0.29 g, 0.775 mmol) were dissolved in 4 mL THF. The mixture was stirred at room temperature for 30 min. The compound **3** (0.15 g, 0.387 mmol) was added to the mixture and stirred at room temperature for 1 h. Then the reaction solution was extracted with EA three times. The combined extract was dried over anhydrous Na_2SO_4. After filtration, the solvent was evaporated under reduced pressure, and the residue was purified on silica gel by flash column chromatography (CH_2Cl_2/MeOH, 40:1, vol/vol) giving compound **4** (113 mg, 55.8%) as white solid. ^{1}H NMR (300 MHz, $CDCl_3$) δ 8.83 (s, 1 H), 7.95 (s, 2 H), 7.78 (s, 2 H), 7.42-7.37 (t, J = 7.5 Hz, 1 H), 7.15-7.13 (d, J = 6.0 Hz, 1 H), 6.80-6.73 (m, 2 H), 4.78-4.74 (d, J = 12.0 Hz, 1 H), 4.66-4.62 (d, J = 12.0 Hz, 1 H), 3.92 (s, 3 H), 3.27-3.22 (t, J = 7.5 Hz, 2 H), 3.03 (s, 3 H), 2.38-2.33 (t, J = 7.5 Hz, 2 H), 1.69-1.55 (m, 4 H), 1.44-1.36 (m, 2 H). ^{13}C NMR (300 MHz, $CDCl_3$) δ 182.66, 171.80, 167.41, 166.21, 164.08, 163.47, 160.54, 138.68, 133.77, 129.81, 127.99, 126.42, 122.77, 121.68, 121.43, 107.82, 107.54, 100.42, 100.08, 59.47, 56.35, 51.28, 39.41, 37.91, 28.66, 26.36, 25.13. HR-MS (ESI) m/z: calculated for $C_{24}H_{27}N_8O_3S$ $[M+H]^+$: 527.1984, found 527.1994.

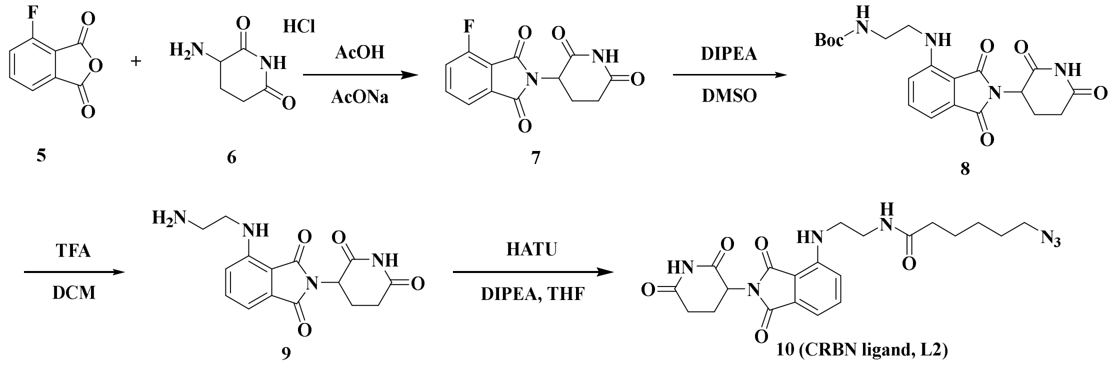

General procedure for the synthesis of compound 7. 3-Fluorophthalic anhydride (25.0 g, 151.00 mmol) and 3-amino-2,6-piperidinedione hydrochloride (25.0 g, 151.00 mmol) were weighed into the reaction flask, followed by Sodium acetate (14.9 g, 181.20 mmol), 300 mL of acetic acid was added into the reaction flask. The temperature of the reaction solution was raised to 120 °C, and the reaction was stirred for 6 h. After cooling, the mixture was concentrated in vacuo. Pouring water into the reaction flask and solid was precipitated, and filtrated to give compound **7** (30 g, 72.2%) as a purple solid. ^{1}H NMR (300 MHz, DMSO-d_6) δ 11.18 (s, 1 H), 7.99-7.92 (m, 1 H), 7.81-7.71 (s, 2 H), 5.21-5.15 (dd, J1 = 15.0 Hz, J2 = 6.0 Hz, 1 H), 2.97-2.85 (m, 1 H),2.65-2.46 (m, 2 H), 2.10-2.06 (t, J = 6.0 Hz, 1 H). ^{13}C NMR (75 MHz, DMSO-d_6) δ 172.78, 169.73, 166.14, 163.99, 158.56, 155.08, 138.12, 138.01, 133.45, 123.14, 122.88, 120.08, 49.09, 30.91, 21.84. HR-MS (ESI) m/z: calculated for $C_{13}H_{10}FN_2O_4$ $[M+Na]^+$: 299.0439, found 299.0458.

General procedure for the synthesis of compound 8. To the solution of compound **7** (10.7 g, 39.00 mmol) in DMSO were added N-Boc-ethylenediamine (6.8 g, 43.00 mmol), DIPEA (15.0 g, 116.00 mmol). The reaction solution was stirred at 60 °C for 2 h. Then the reaction solution was cooled to room temperature and extracted with DCM three times. The combined extract was dried over anhydrous Na_2SO_4. After filtration, the solvent was evaporated under reduced pressure, and the residue was purified on silica gel by flash column chromatography (petroleum ether/ethyl estate, 1:1, vol/vol) giving compound 8 (9.7 g, 59.9%) as yellow solid. ^{1}H NMR (300 MHz, DMSO-d_6) δ 11.11 (s, 1 H), 7.61-7.55 (t, J = 9.0 Hz,1 H), 7.16-7.13 (d, J = 9.0 Hz,1 H), 7.04-7.02 (d, J = 6.0 Hz, 2 H), 6.72-6.70 (d, J = 6.0 Hz, 1 H), 5.09-5.03 (dd, J1 = 12.0 Hz, J2 = 6.0 Hz, 1 H), 3.38-3.35 (d, J = 9.0 Hz, 2 H), 3.14-3.12 (d, J = 6.0 Hz, 2 H), 2.96-2.84 (m, 1 H), 2.62-2.47 (m, 2 H), 2.04-1.99 (t, J = 7.5 Hz, 1 H), 1.37 (s, 9 H). ^{13}C NMR (75 MHz, DMSO-d_6) δ 172.82, 170.07, 168.69, 167.29, 155.87, 146.37, 136.15, 132.19, 117.04, 110.47, 109.18, 77.76, 48.47, 41.53, 30.95, 28.16, 22.14. HR-MS (ESI) m/z: calculated for $C_{20}H_{24}N_4O_6$ $[M+Na]^+$: 439.1588, found 439.1547.

General procedure for the synthesis of compound 9. The compound **8** (9.7 g, 23.32 mmol) was dissolved in 100 mL DCM and stirred in an ice bath. Trifluoroacetic acid (26.6 g, 233.20 mmol) was added to the reaction solution drop by drop. The reaction was stirred at room temperature for 2 h and concentrated to dryness, giving 7.2 g crude product as yellow solid. ^{1}H NMR (300 MHz, DMSO-d_6) δ 11.13 (s, 1 H), 7.98 (s, 3 H), 7.65-7.62 (d, J = 9.0 Hz,1 H), 7.21-7.18 (d, J = 9.0 Hz, 3 H), 7.10-7.08 (d, J = 6.0 Hz, 1 H), 6.87-6.83 (d, J = 6.0 Hz, 1 H), 5.11-5.05 (dd, J1 = 12.0 Hz, J2 = 6.0 Hz, 1 H), 3.63-3.57 (dd, J1 = 12.0 Hz, J2 = 6.0 Hz, 2 H), 3.02-2.85 (m, 3 H), 2.63-2.46 (m, 2 H), 2.06-2.00 (m, 1 H). ^{13}C NMR (75 MHz, DMSO-d_6) δ 173.34, 170.58, 169.07, 167.75, 146.27, 136.77, 132.77, 117.62, 111.51, 110.53, 49.03, 38.22, 31.46, 22.65. HR-MS (ESI) m/z: calculated for $C_{15}H_{16}N_4O_4$ $[M+H]^+$: 317.1244, found 317.1251.

General procedure for the synthesis of compound 10 (CRBN ligand, L2). To a 25 mL three-neck

round-bottom flask containing a magnetic stir bar was added compound **9** (0.50 g, 1.02 mmol), 6 mL of THF and trimethylsilylacetylene (TMSA, 0.14 g, 1.22 mmol). The mixture was evacuated three times under an N_2 atmosphere. Then tetrabutylammonium fluoride (TBAF, 1.53 mL, 1.22 mmol) was added to the mixture and stirred at 65 °C for 3 h. Then the reaction solution was cooled to room temperature and extracted with EA for three times. The organic layer was separated, concentrated to dryness and used directly in the next step without purification. ^{1}H NMR (300 MHz, DMSO-d$_6$) δ 11.12 (s, 1 H), 8.07-8.03 (t, J = 6.0 Hz,1 H), 7.62-7.57 (d, J = 7.5 Hz,1 H), 7.20-7.17 (d, J = 9.0 Hz, 1 H), 7.05-7.03 (d, J = 6.0 Hz, 1 H), 6.75-6.71 (t, J = 6.0 Hz, 1 H), 5.10-5.04 (dd, J1 = 12.0 Hz, J2 = 6.0 Hz, 1 H), 3.40-3.25 (m, 8 H), 2.97-2.84 (m, 1 H), 2.63-2.52 (m, 1 H), 2.10-2.05 (t, J =7.5 Hz, 2 H), 1.56-1.46 (m, 4 H), 1.32-1.25 (m, 2 H). ^{13}C NMR (300 MHz, DMSO) δ 172.96, 172.71, 170.23, 168.85, 167.44, 146.48, 136.32, 132.33, 117.28, 110.66, 109.35, 50.61, 48.64, 41.56, 40.44, 40.16, 38.09, 35.30, 31.10, 28.12, 25.87, 24.83, 22.28. HR-MS (ESI) m/z: calculated for $C_{21}H_{25}N_7O_5$ $[M+H]^+$: 456.1990, found 456.1952.

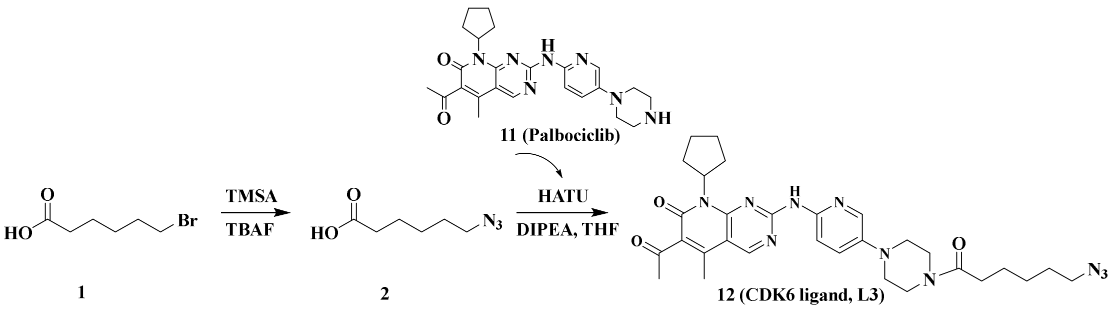

General procedure for the synthesis of compound 12 (CDK6 ligand, L3). The compound **2** (0.117 g, 0.72 mmol), DIPEA (0.320 g, 2.48 mmol) and HATU (0.230 g, 0.60 mmol) were dissolved in 6 mL THF. The mixture was stirred at room temperature for 10 min. The compound **11** (0.220 g, 0.50 mmol) was added to the mixture and stirred at room temperature for 1 h. Then the reaction solution was extracted with DCM for three times. The combined extract was dried over anhydrous Na_2SO_4. After filtration the solvent was evaporated under reduced pressure, and the residue was purified on silica gel by flash column chromatography (CH_2Cl_2/MeOH, 80:1, vol/vol) giving compound **12** (220 mg, 78.6%) as yellow solid. ^{1}H NMR (300 MHz, $CDCl_3$) δ 9.41 (s, 1 H), 8.94 (s, 1 H), 8.23-8.20 (d, J = 9.0 Hz, 1 H), 8.10 (s, 1 H), 7.37-7.25 (m, 1 H), 5.92-5.86 (t, J = 9.0 Hz, 1 H), 3.82 (s, 2 H), 3.67 (s, 2 H), 3.46-3.42 (t, J = 6.0 Hz, 2 H), 3.30-3.17 (m, 4 H), 2.55-2.52 (d, J = 9.0 Hz, 3 H), 2.39 (s, 7 H), 2.07 (s, 2 H), 1.91-1.89 (d, J = 9.0 Hz, 3 H), 1.71-1.69 (m, 5 H), 1.55-1.53 (m, 2 H). ^{13}C NMR (300 MHz, $CDCl_3$) δ 202.69, 171.23, 161.38, 158.07, 157.32, 155.55, 145.73, 143.11, 141.84, 136.89, 130.73, 126.75, 113.62, 107.64, 54.15, 51.27, 50.05, 49.75, 45.32, 41.33, 33.74, 32.95, 32.52, 31.56, 28.74, 28.06, 27.95, 26.57, 25.75, 24.69, 24.32, 13.99. HR-MS (ESI) m/z: calculated for $C_{30}H_{38}N_{10}O_3$ $[M+H]^+$: 587.3201, found 587.3208.

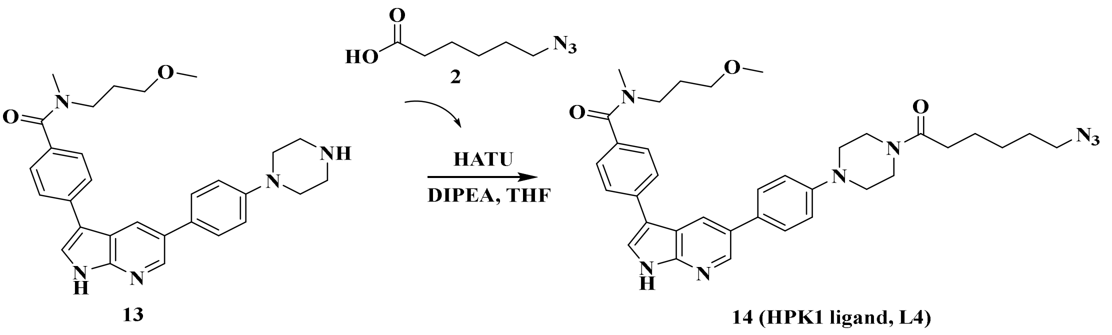

General procedure for the synthesis of compound 14 (HPK1 ligand, L4). The intermediate **2** (0.117 g, 0.72 mmol), DIPEA (0.320 g, 2.48 mmol) and HATU (0.230 g, 0.60 mmol) were dissolved in 6 mL THF. The mixture was stirred at room temperature for 10 min. The compound **13** (0.241 g, 0.50 mmol) was added to the mixture and stirred at room temperature for 1 h. Then the reaction solution was extracted with DCM for three times. The combined extract was dried over anhydrous Na_2SO_4. After filtration the solvent was evaporated under reduced pressure, and the residue was purified on silica gel by flash column chromatography ($CH2Cl_2$/MeOH, 80:1, vol/vol) giving compound **14** (200 mg, 64.3%) as white solid. ^{1}H NMR (300 MHz, $CDCl_3$) δ (ppm) = 11.13 (s, 1H), 8.59 (s, 1H), 8.36 (s, 1H), 7.69 (d, J = 6.0 Hz, 2H), 7.58 (t, J = 6.0 Hz, 3H), 7.51 (d, J = 9.0 Hz, 2H), 7.04 (d, J = 9.0 Hz, 2H), 3.82 (t, J = 4.5 Hz, 2H), 3.68-3.58 (m, 3H), 3.51-3.41 (m, 2H), 3.38-3.35 (m, 2H), 3.32-3.21 (m, 8H), 3.09 (s, 3H), 2.41 (t, J = 6.0 Hz, 2H), 1.98-1.87 (m, 2H), 1.77-1.81 (m, 4H), 1.51-1.43 (m, 2H). ^{13}C NMR (75 MHz, $CDCl_3$) δ (ppm) = 171.30, 150.25, 148.16, 141.92, 136.25, 134.36, 131.23,130.09, 128.26, 127.74, 126.84, 126.46, 123.63, 118.71, 116.98, 115.86, 58.70, 51.32, 49.63, 49.31, 45.45, 41.46, 33.04, 28.79, 26.61, 24.77. HRMS (ESI) m/z: calculated for $C_{35}H_{42}N_8O_3[M+H]^+$: 623.3458; found, 623.3450.

^{1}H NMR Spectra

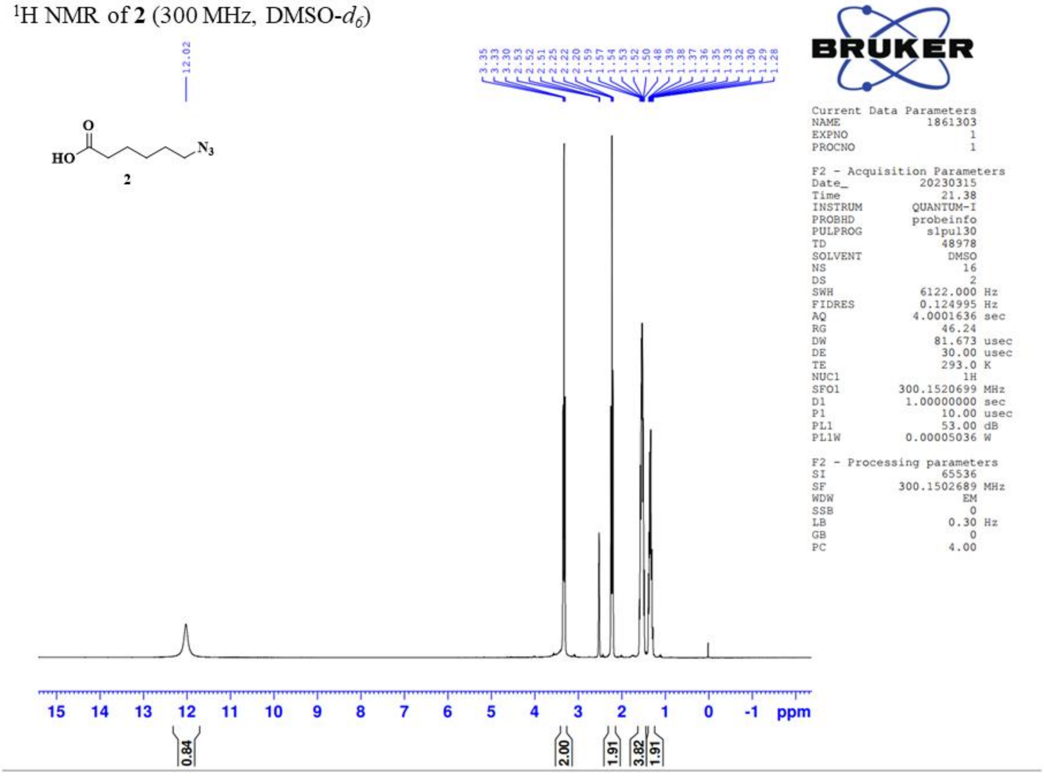

Supplementary Fig. 27. ^{1}H NMR spectrum of compound **2** (DMSO-d_6).

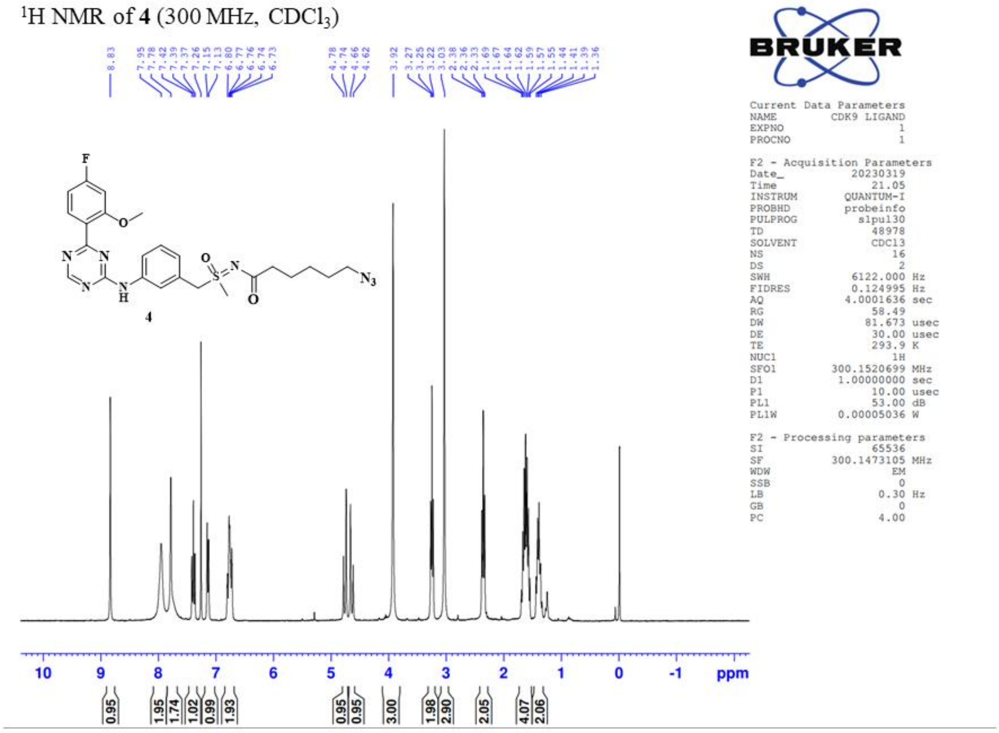

Supplementary Fig. 28. ^{1}H NMR spectrum of compound **4** ($CDCl_3$).

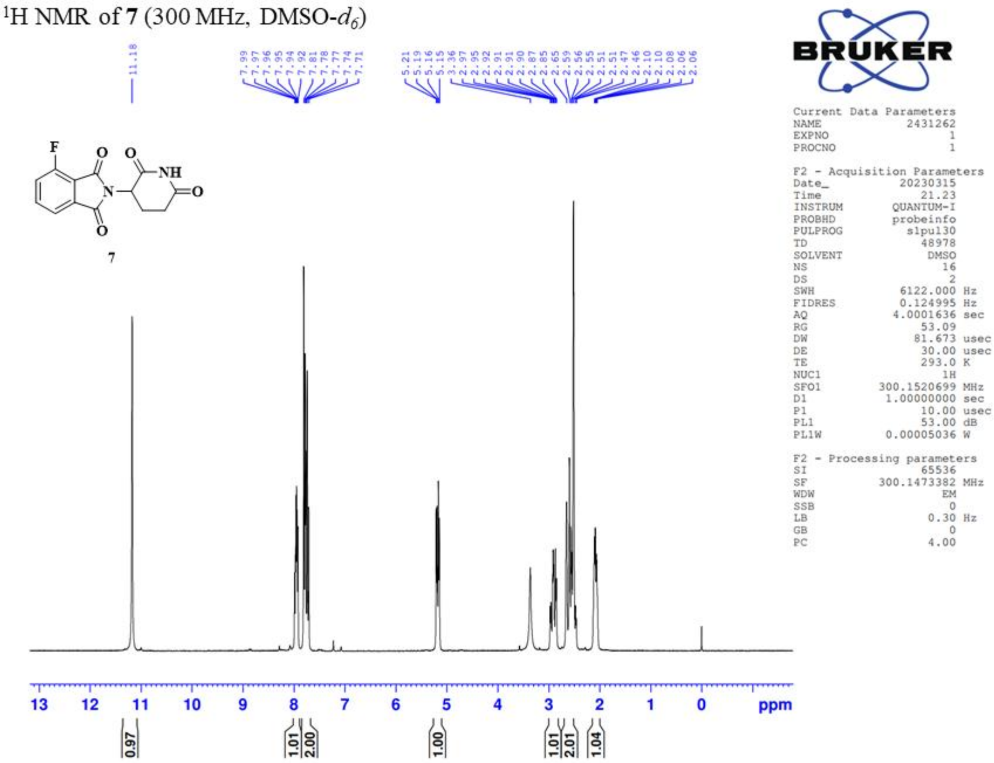

Supplementary Fig. 29. ^{1}H NMR spectrum of compound **7** (DMSO-d_6).

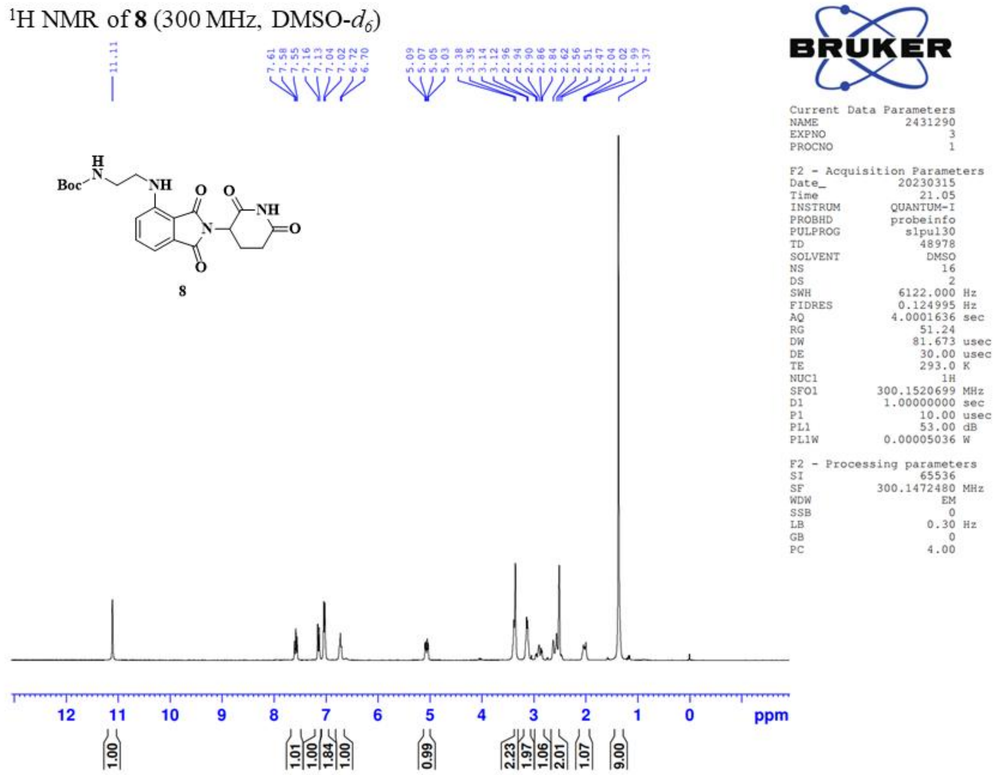

Supplementary Fig. 30. ^{1}H NMR spectrum of compound **7** (DMSO-d_6).

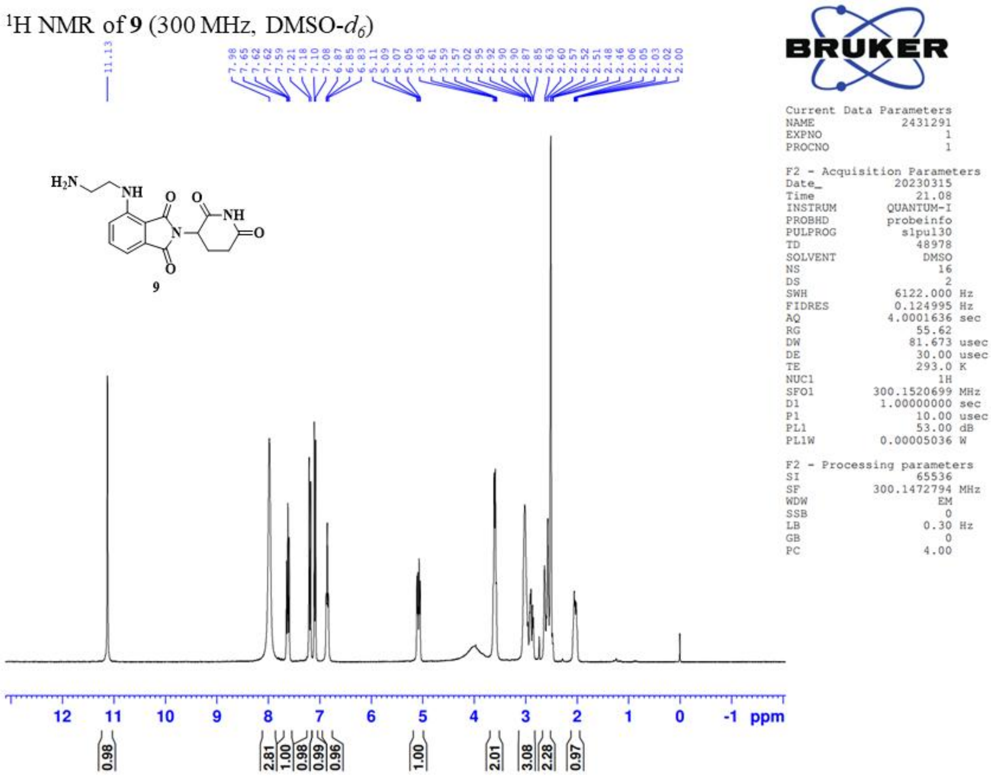

Supplementary Fig. 31. ^{1}H NMR spectrum of compound **9** (DMSO-d_6).

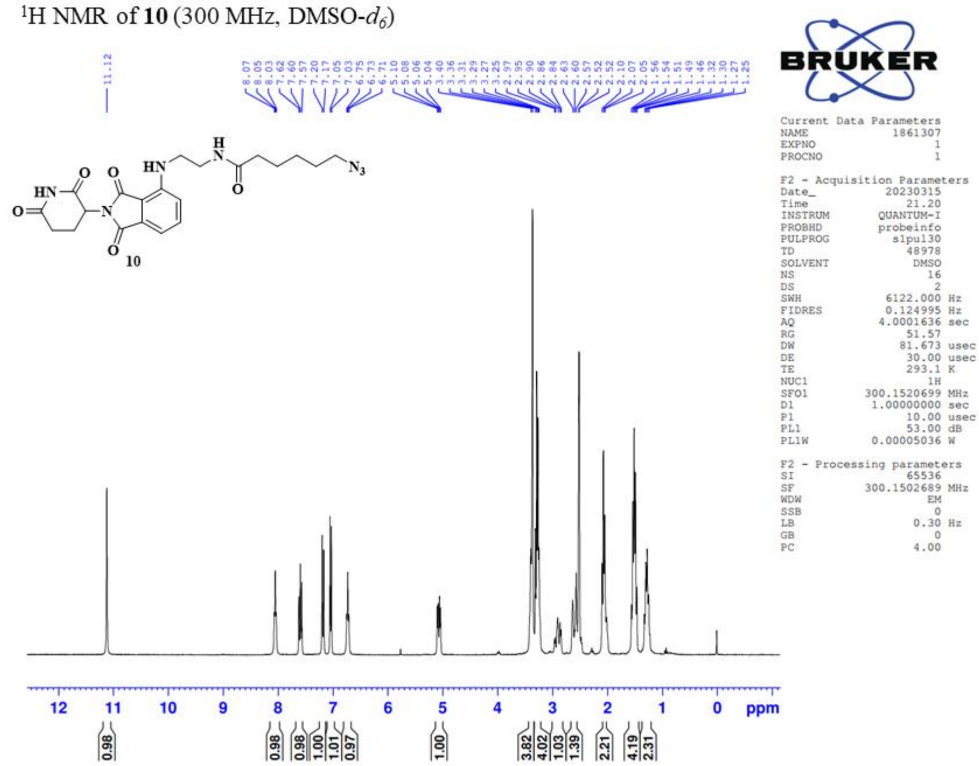

Supplementary Fig. 32. ^{1}H NMR spectrum of compound **10** (DMSO-d_6).

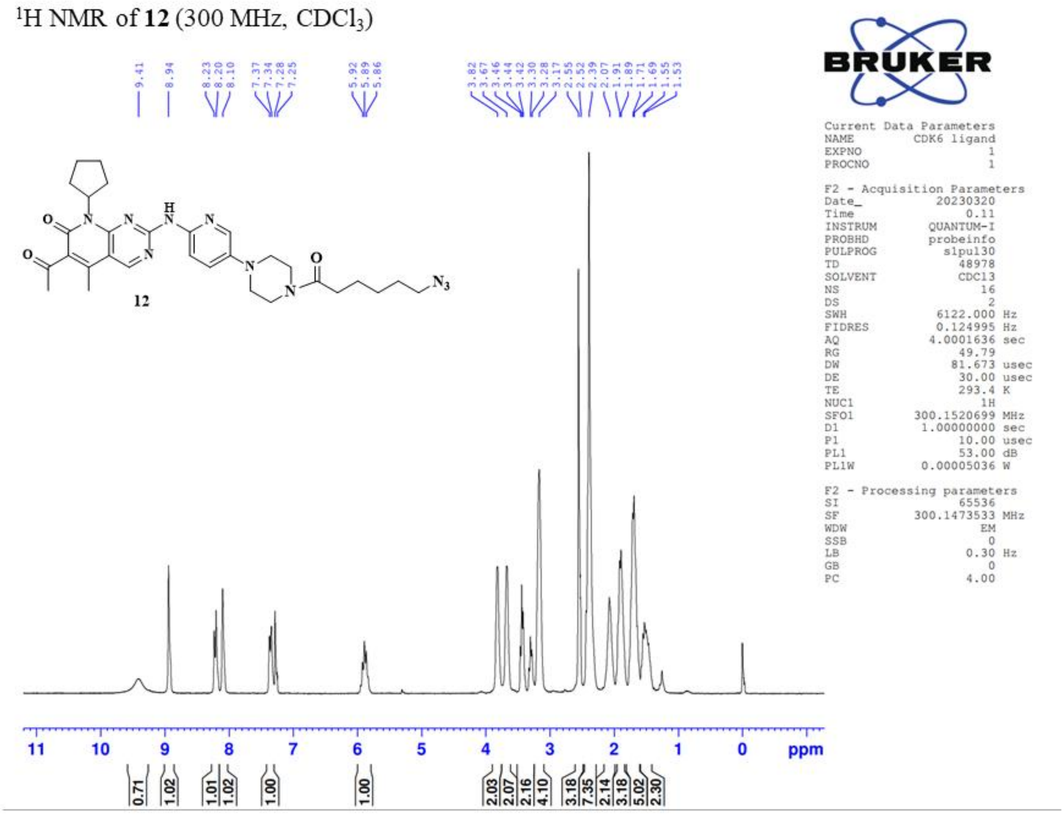

Supplementary Fig. 33. ^{1}H NMR spectrum of compound **12** (CDCl$_3$).

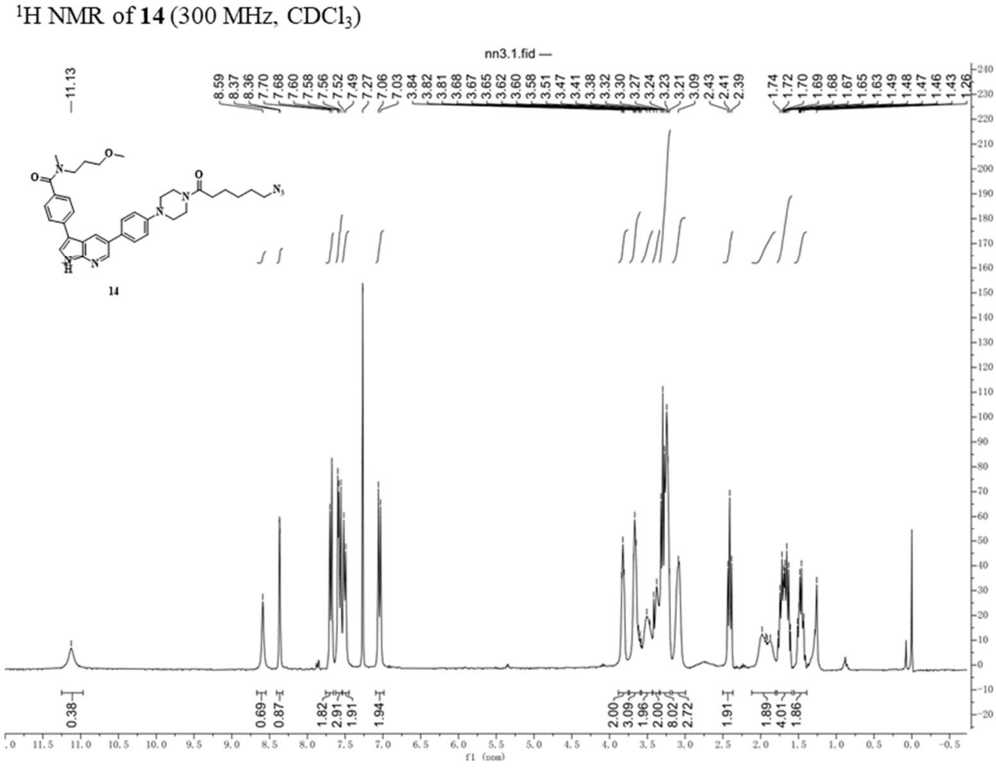

Supplementary Fig. 34. ^{1}H NMR spectrum of compound **14** (CDCl$_3$).

^{13}C NMR Spectra

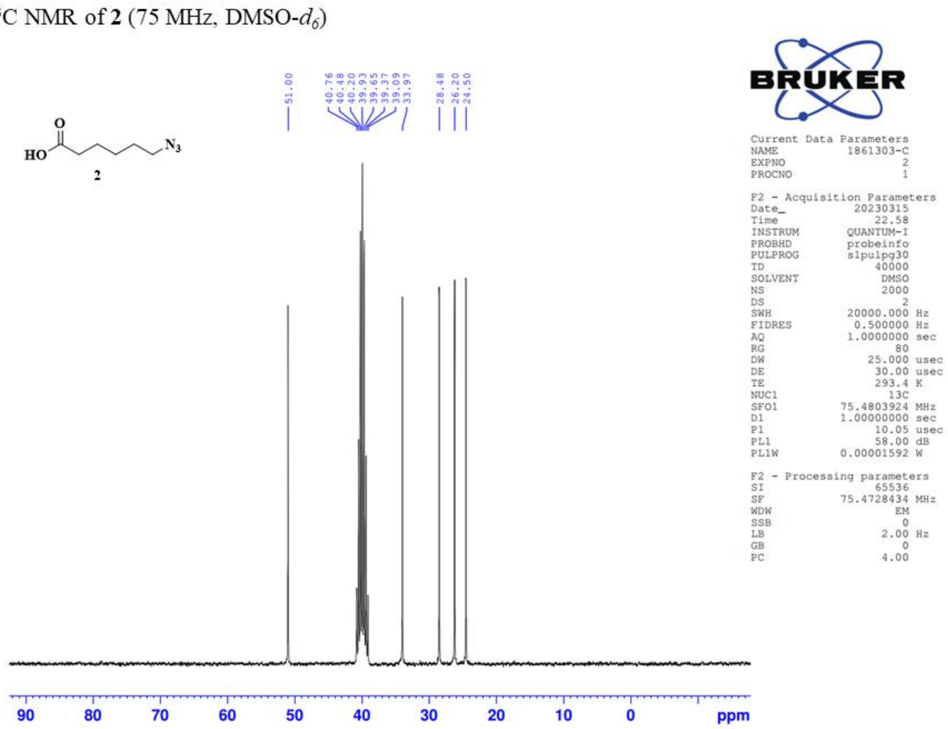

Supplementary Fig. 35. ^{1}C NMR spectrum of compound **2** (DMSO-d_6).

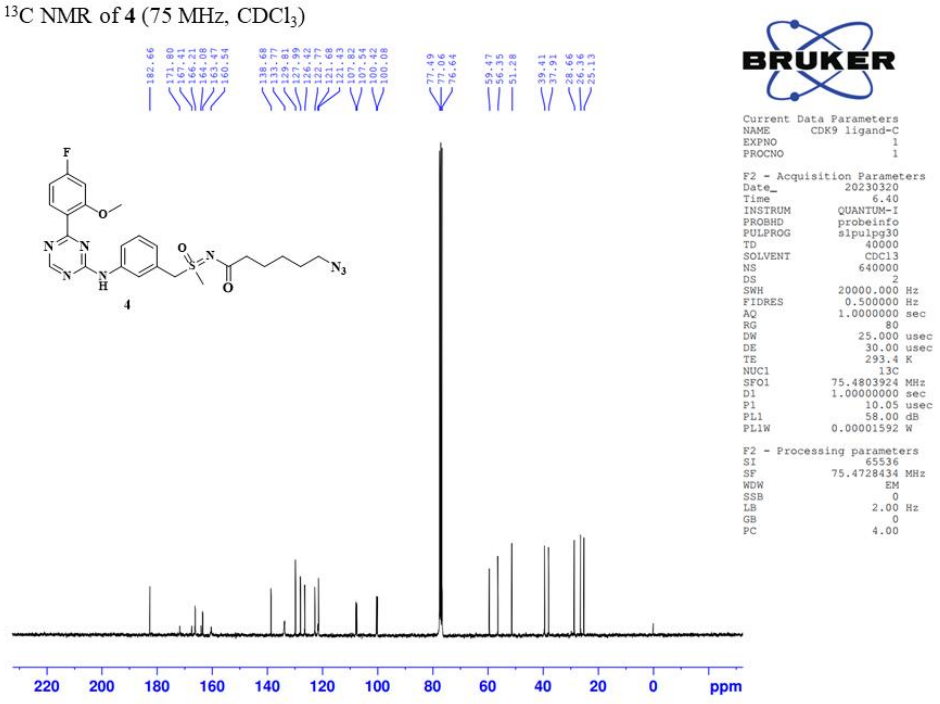

Supplementary Fig. 36. ^{1}C NMR spectrum of compound **4** ($CDCl_3$).

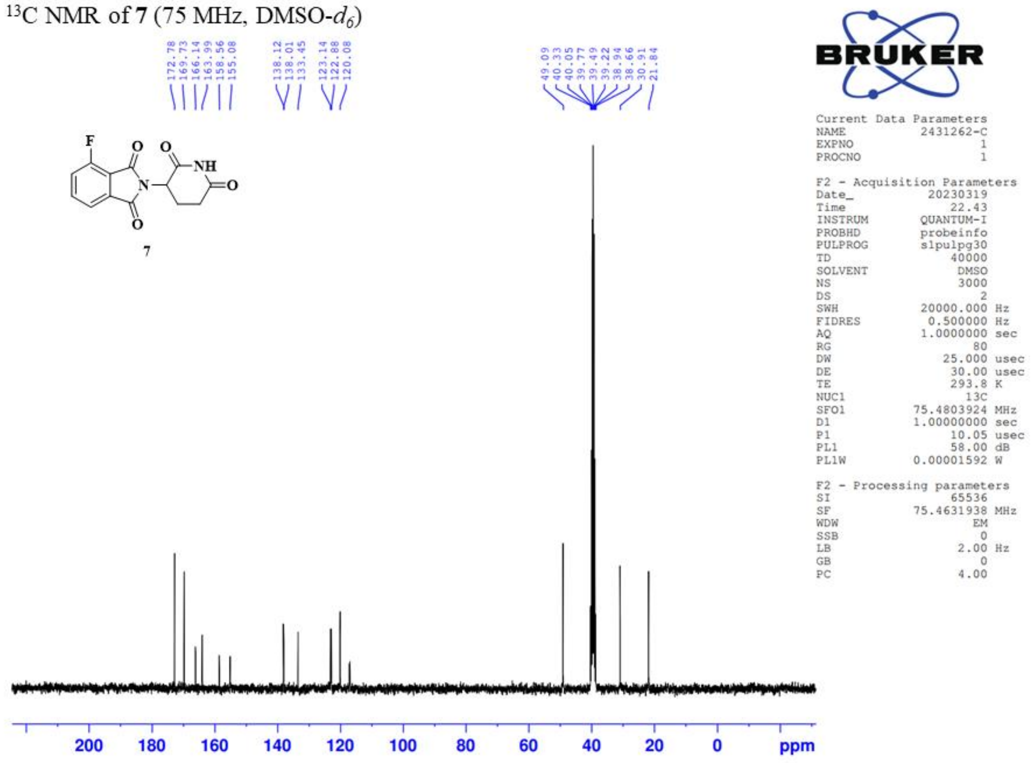

Supplementary Fig. 37. [1]C NMR spectrum of compound **7** (DMSO-d_6).

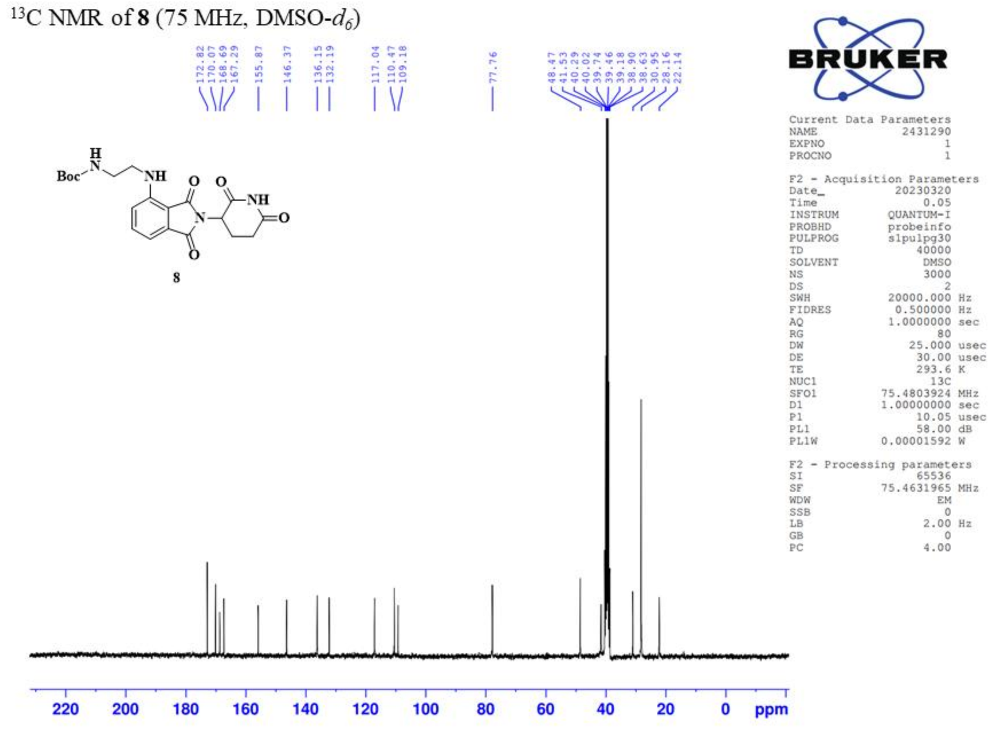

Supplementary Fig. 38. [1]C NMR spectrum of compound **8** (DMSO-d_6).

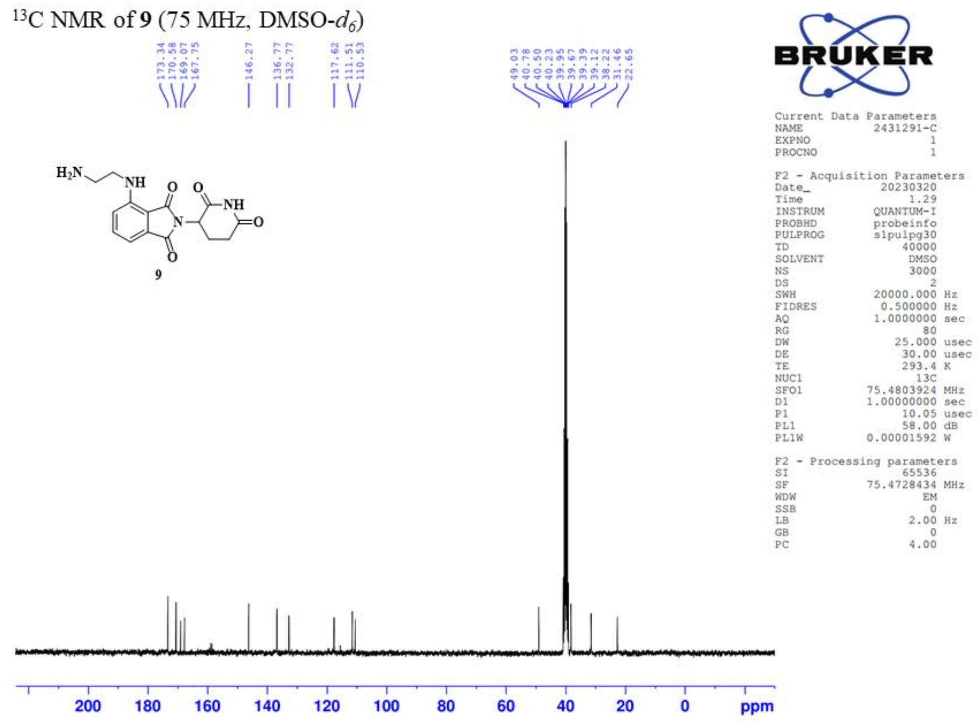

Supplementary Fig. 39. ^{1}C NMR spectrum of compound **9** (DMSO-d_6).

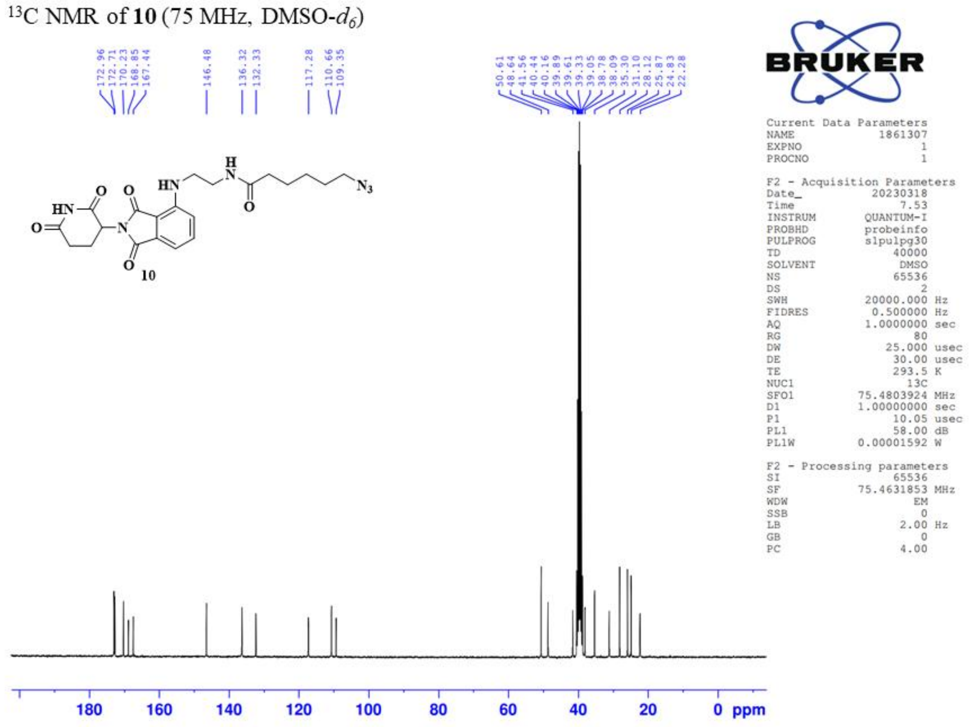

Supplementary Fig. 40. ^{1}C NMR spectrum of compound **10** (DMSO-d_6).

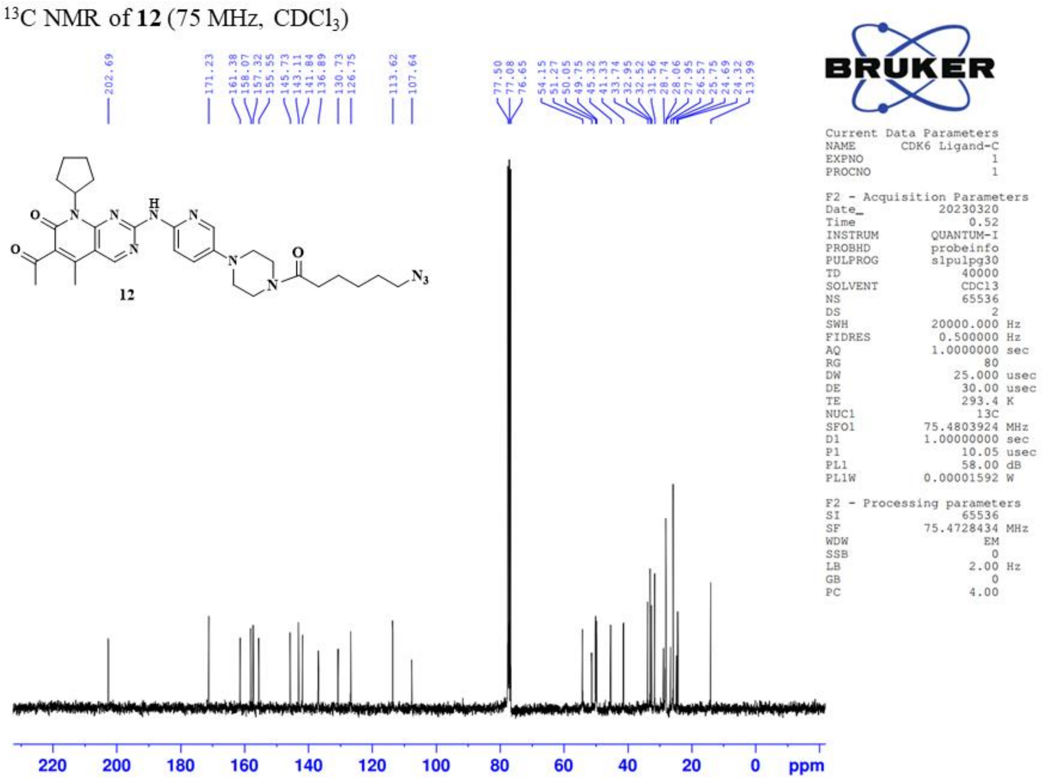

Supplementary Fig. 41. ^{1}C NMR spectrum of compound **12** (CDCl$_3$).

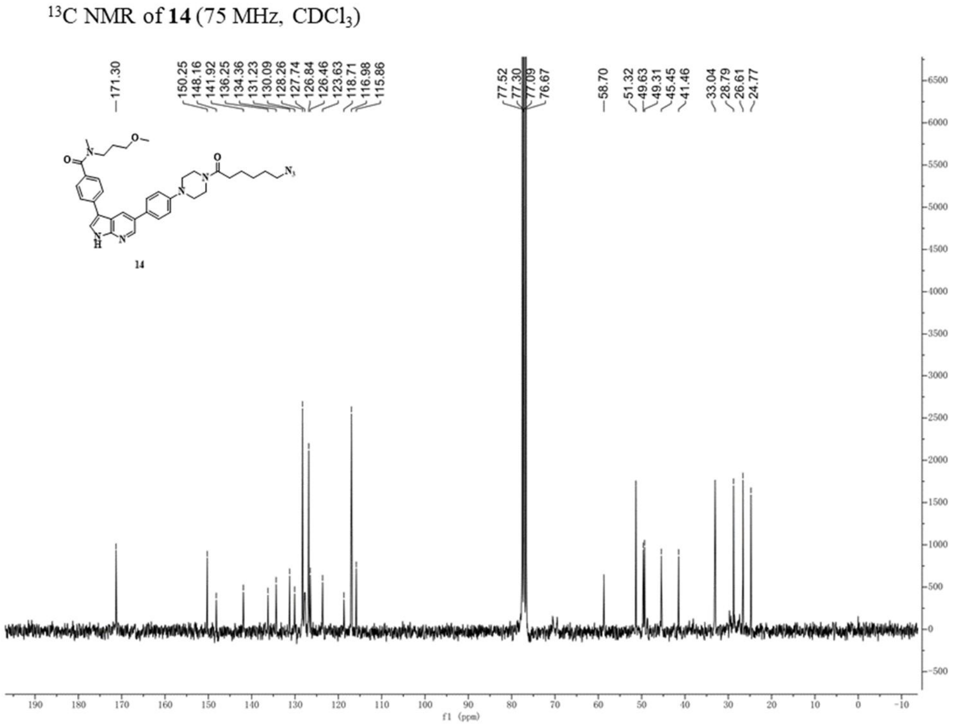

Supplementary Fig. 42. ^{1}C NMR spectrum of compound **14** (CDCl$_3$).

HRMS Spectra

HRMS spectrum of **2** (ESI$^-$)

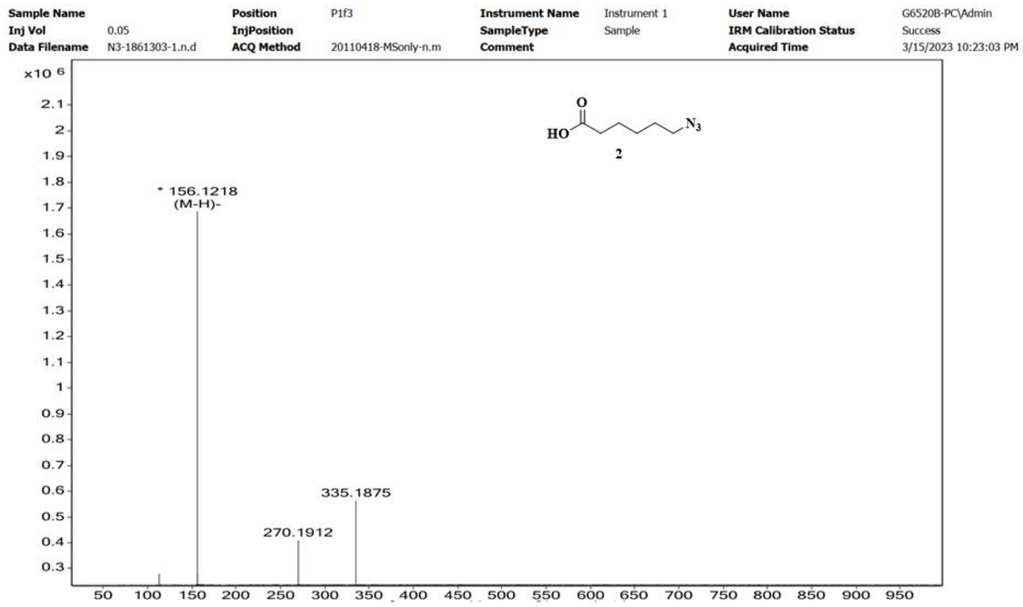

Supplementary Fig. 43. HRMS spectrum of compound 2.

HRMS spectrum of **4** (ESI$^+$)

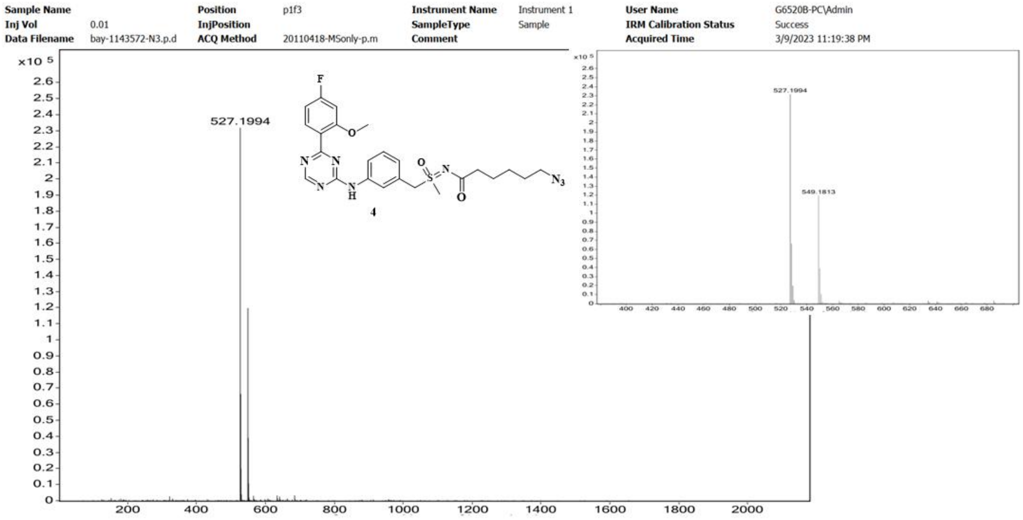

Supplementary Fig. 44. HRMS spectrum of compound **4**.

HRMS spectrum of **7** (ESI$^+$)

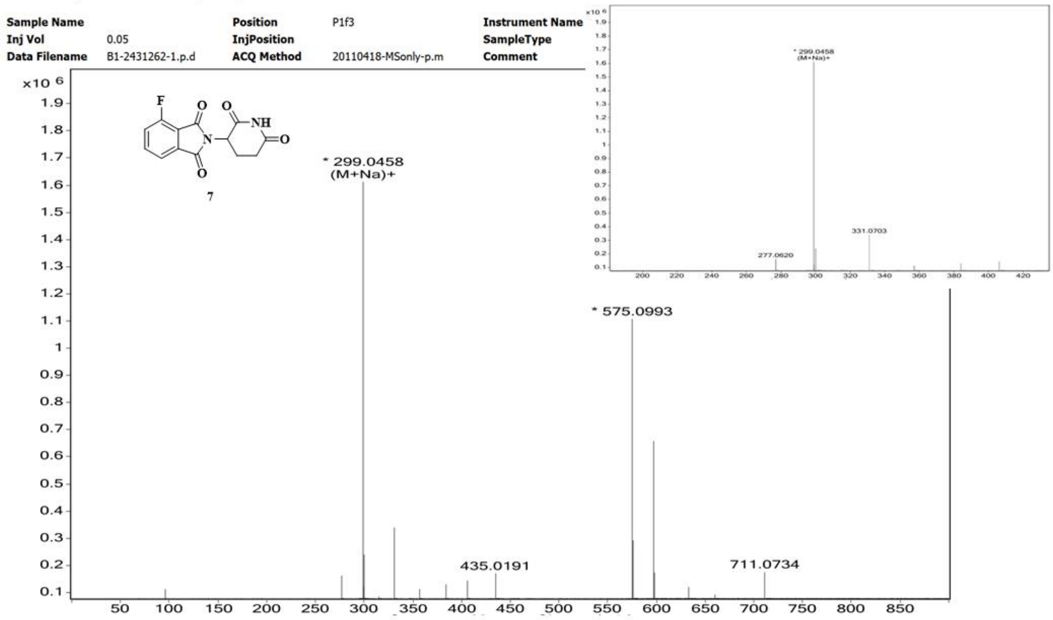

Supplementary Fig. 45. HRMS spectrum of compound **7**.

HRMS spectrum of **8** (ESI$^+$)

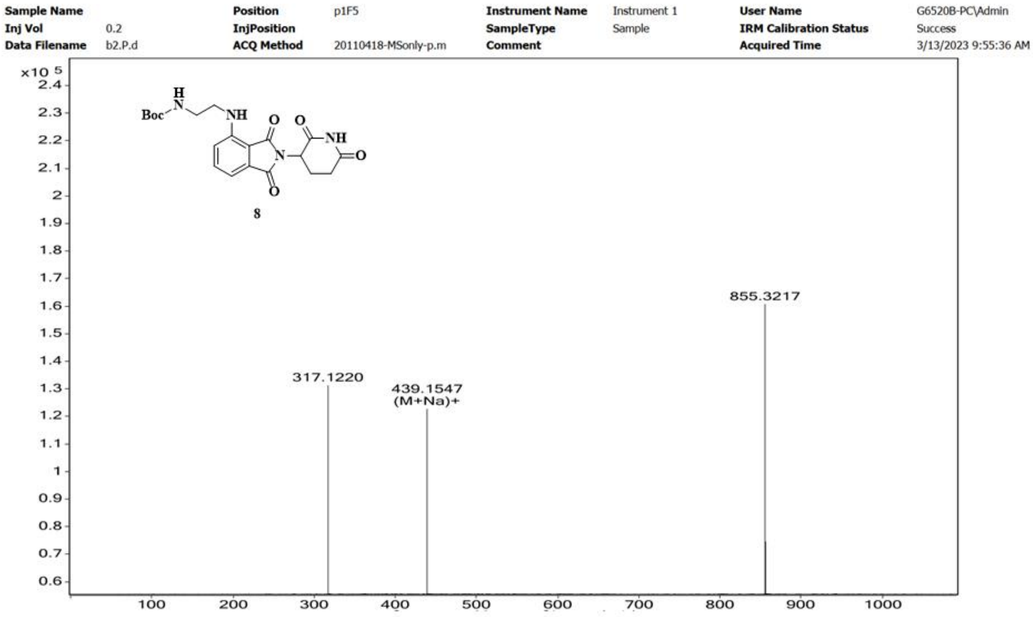

Supplementary Fig. 46. HRMS spectrum of compound **8**.

HRMS spectrum of **9** (ESI⁺)

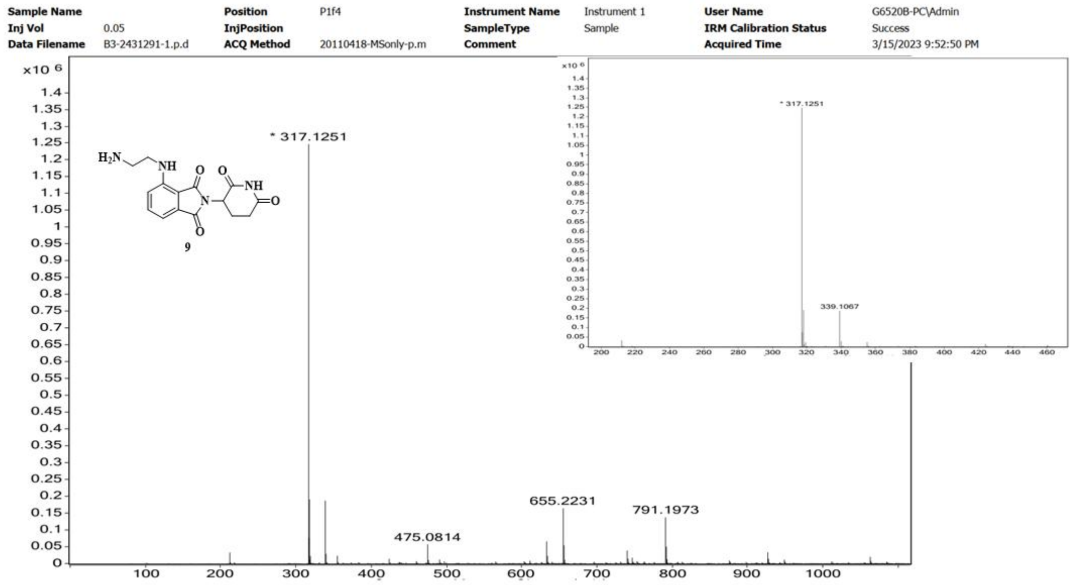

Supplementary Fig. 47. HRMS spectrum of compound **9**.

HRMS spectrum of **10** (ESI⁺)

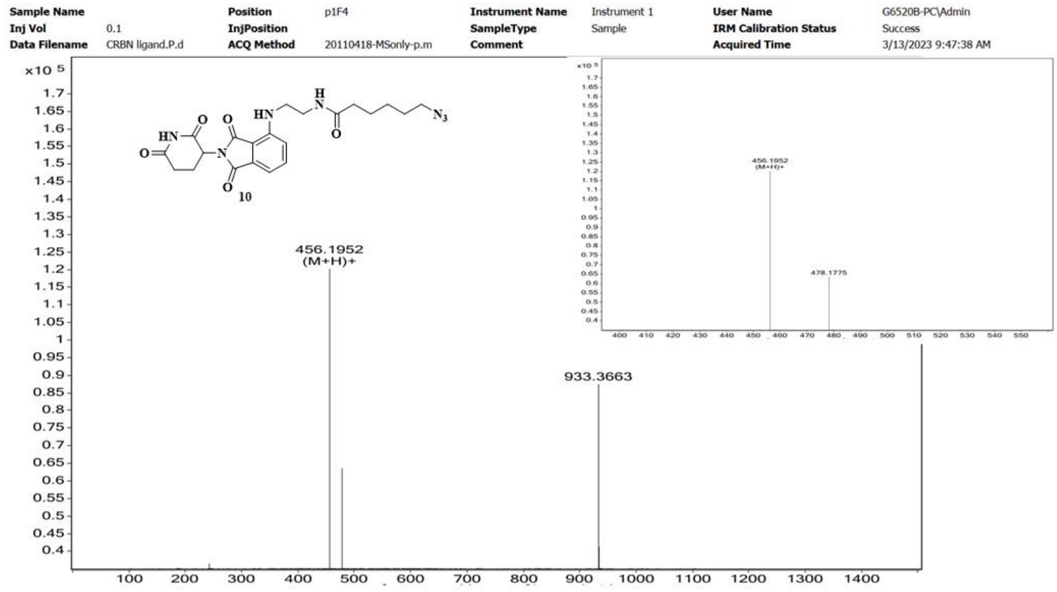

Supplementary Fig. 48. HRMS spectrum of compound **10**.

HRMS spectrum of **12** (ESI$^+$)

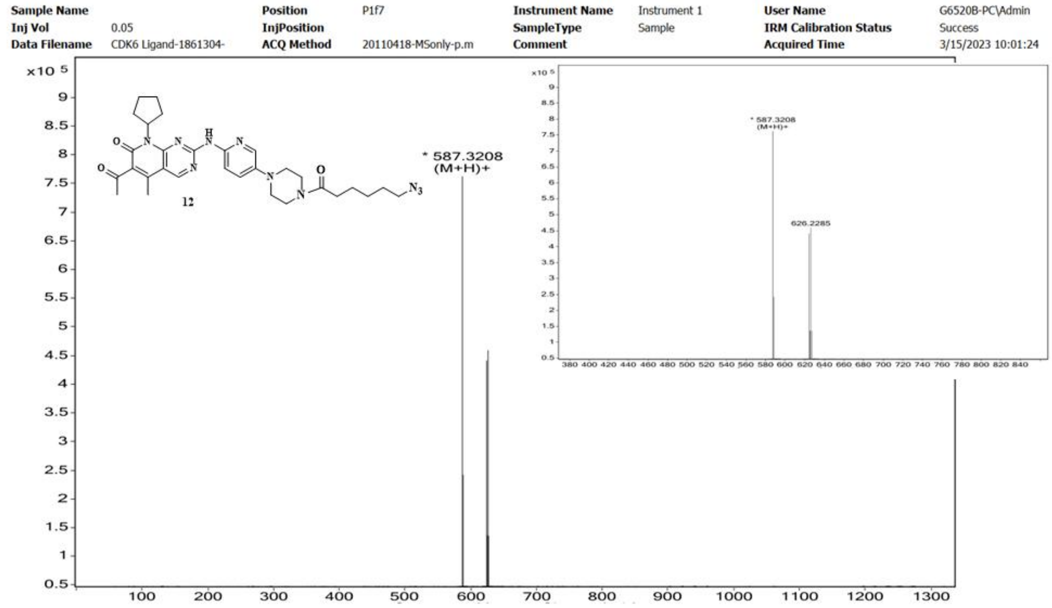

Supplementary Fig. 49. HRMS spectrum of compound **12**.

HRMS spectrum of **14** (ESI$^+$)

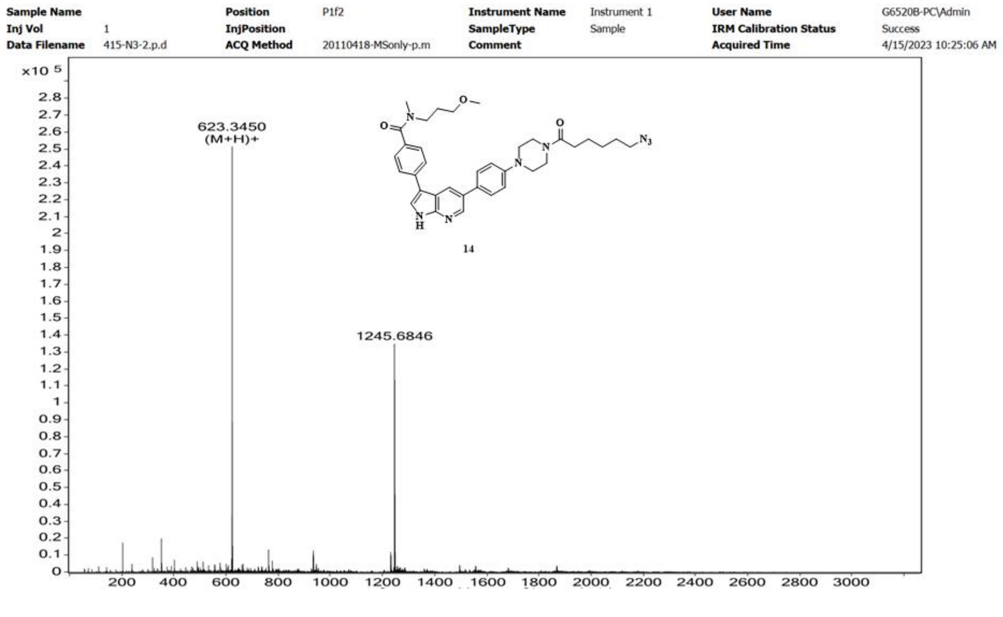

Supplementary Fig. 50. HRMS spectrum of compound **14**.

HPLC Spectra

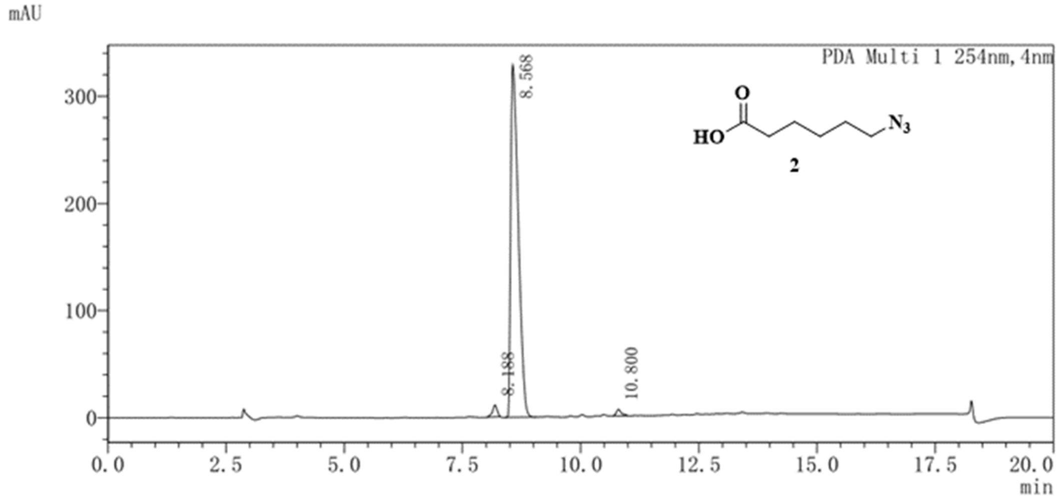

Supplementary Fig. 51. HPLC spectrum of compound **2**.

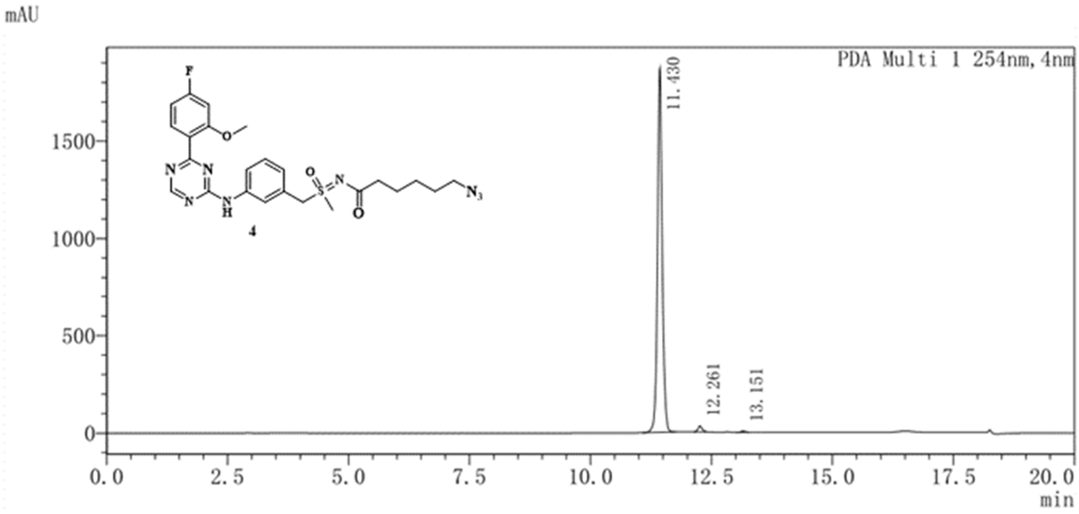

Supplementary Fig. 52. HPLC spectrum of compound **4**.

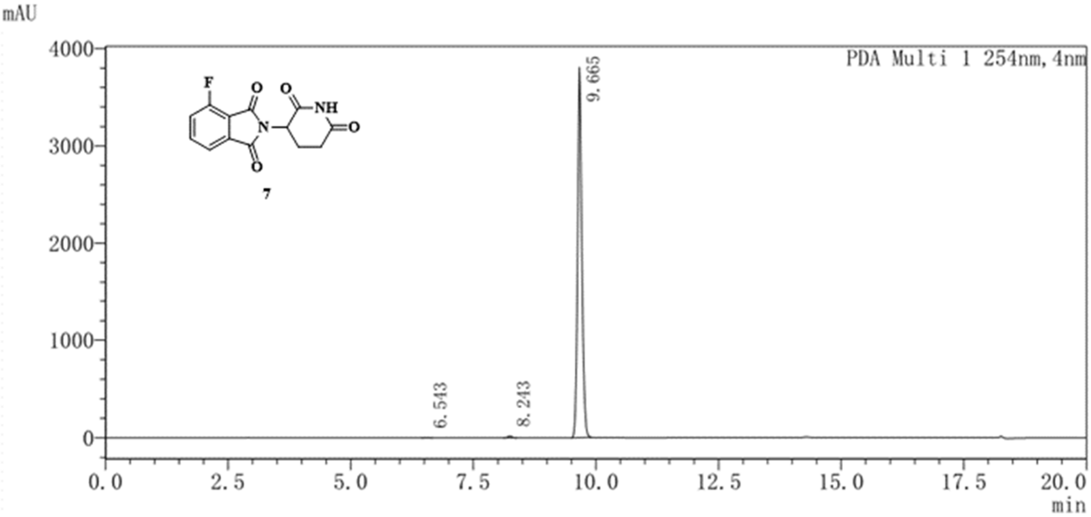

Supplementary Fig. 53. HPLC spectrum of compound **7**.

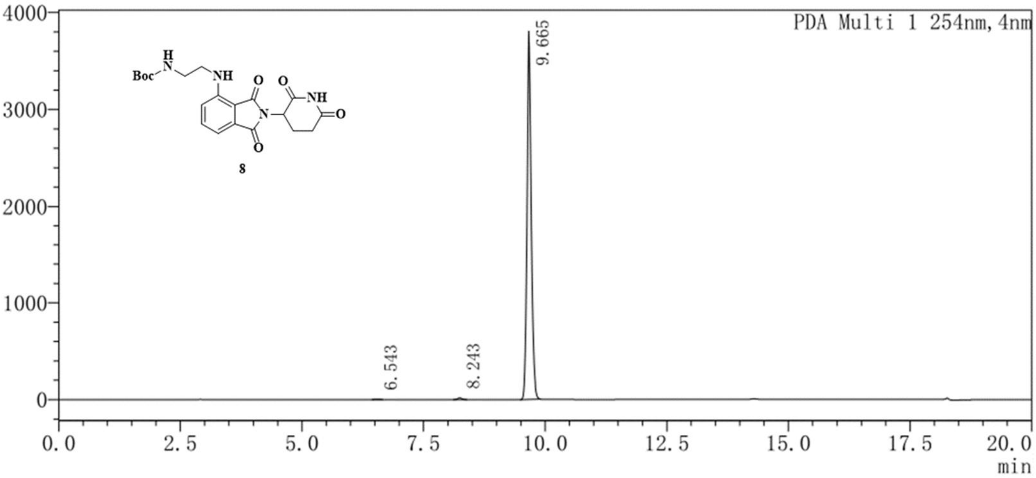

Supplementary Fig. 54. HPLC spectrum of compound **8**.

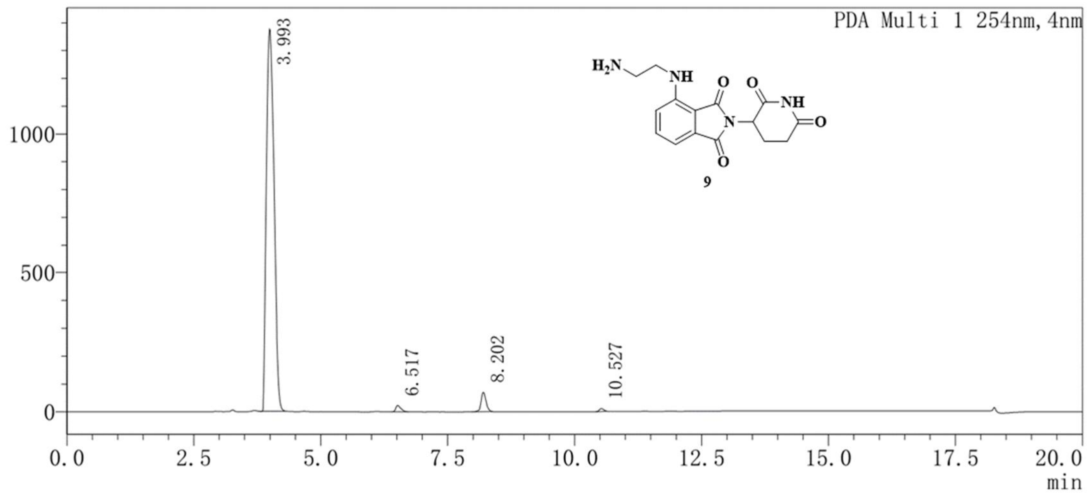

Supplementary Fig. 55. HPLC spectrum of compound **9**.

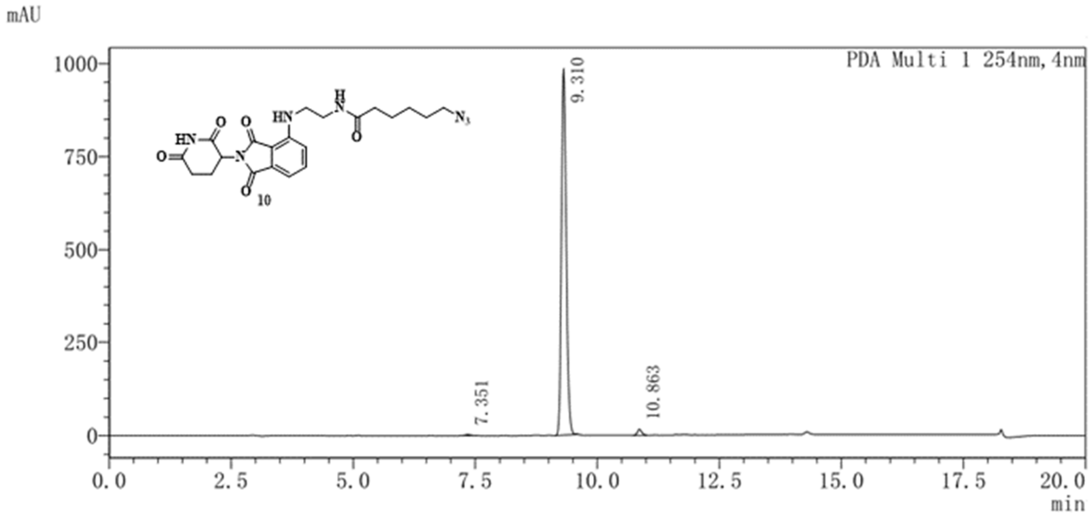

Supplementary Fig. 56. HPLC spectrum of compound **10**.

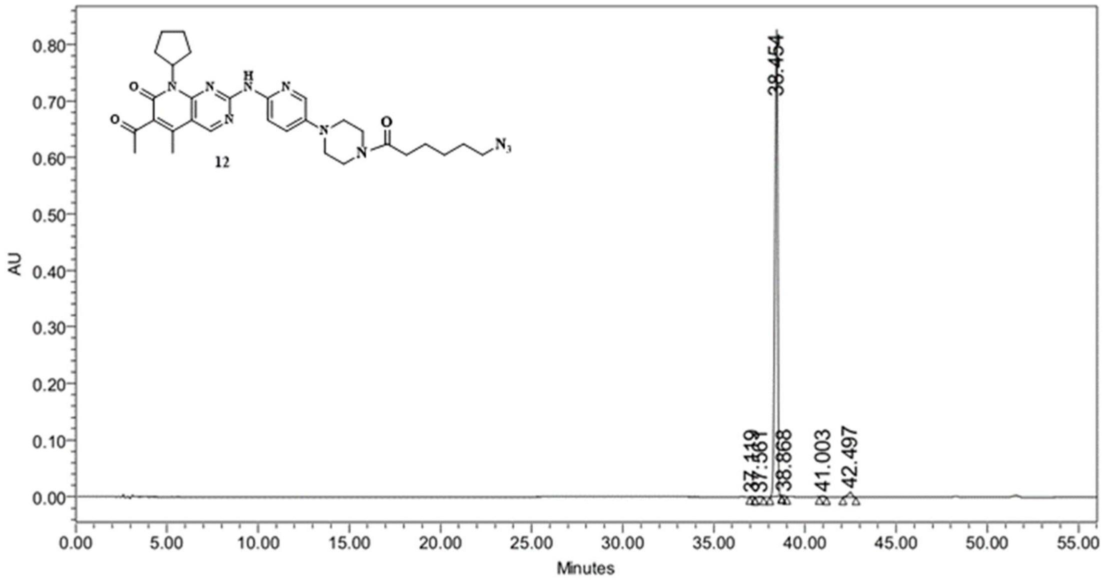

Supplementary Fig. 57. HPLC spectrum of compound **12**.

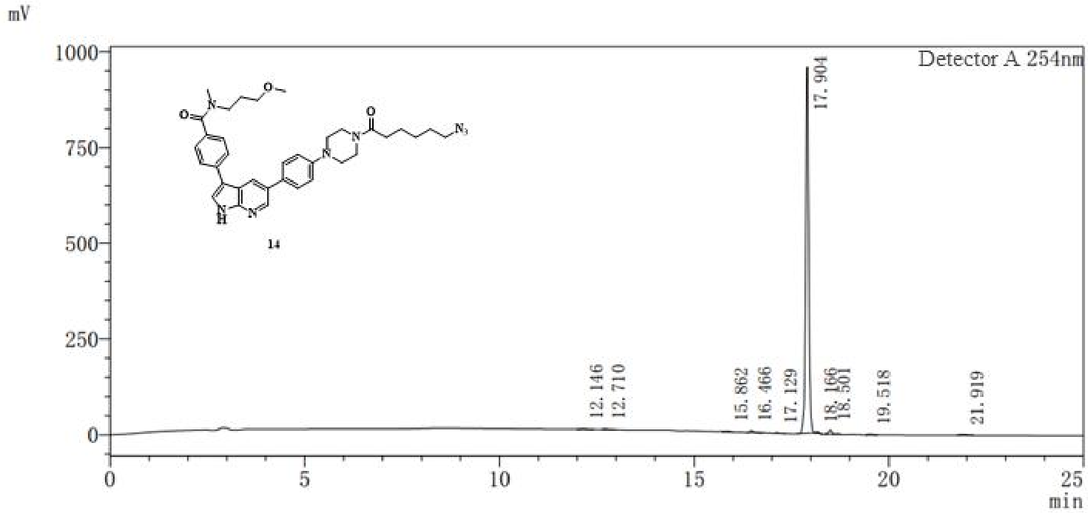

Supplementary Fig. 58. HPLC spectrum of compound **14**.
