# Supplementary material for: DNA framework-engineered chimeras platform enables selectively targeted protein degradation
Source: Nat Commun. 2023 Jul 27;14:4510. doi: 10.1038/s41467-023-40244-7 (PMC10372072; doi:10.1038/s41467-023-40244-7)
Supplement: Supplementary file 3 — Reporting Summary [file 41467_2023_40244_MOESM3_ESM.pdf]

nature portfolio

Corresponding author(s): Yi Ma, Jinlei Bian & Yueqing Gu

Last updated by author(s): Jun 28, 2023

Reporting Summary

Nature Portfolio wishes to improve the reproducibility of the work that we publish. This form provides structure for consistency and transparency in reporting. For further information on Nature Portfolio policies, see our Editorial Policies and the Editorial Policy Checklist.

Statistics

For all statistical analyses, confirm that the following items are present in the figure legend, table legend, main text, or Methods section.

n/a	Confirmed	
☐	☒	The exact sample size (*n*) for each experimental group/condition, given as a discrete number and unit of measurement
☐	☒	A statement on whether measurements were taken from distinct samples or whether the same sample was measured repeatedly
☐	☒	The statistical test(s) used AND whether they are one- or two-sided<br>*Only common tests should be described solely by name; describe more complex techniques in the Methods section.*
☒	☐	A description of all covariates tested
☒	☐	A description of any assumptions or corrections, such as tests of normality and adjustment for multiple comparisons
☐	☒	A full description of the statistical parameters including central tendency (e.g. means) or other basic estimates (e.g. regression coefficient) AND variation (e.g. standard deviation) or associated estimates of uncertainty (e.g. confidence intervals)
☐	☒	For null hypothesis testing, the test statistic (e.g. F, t, r) with confidence intervals, effect sizes, degrees of freedom and P value noted<br>*Give P values as exact values whenever suitable.*
☒	☐	For Bayesian analysis, information on the choice of priors and Markov chain Monte Carlo settings
☒	☐	For hierarchical and complex designs, identification of the appropriate level for tests and full reporting of outcomes
☒	☐	Estimates of effect sizes (e.g. Cohen's d, Pearson's r), indicating how they were calculated

Our web collection on statistics for biologists contains articles on many of the points above.

Software and code

Policy information about availability of computer code

Data collection	Mass spectra of the samples were acquired using LTQ XL liquid mass ion trap mass spectrometer (Thermo, USA). A Bio-Rad imaging system was used for gel scanning. Atomic force microscope image was taken by Burker AXS Dimension Icon. A TEM (JEM-2000 EX II, JEOL Company, USA) was used for imaging of DbTACs and bis-DbTACs equivalents. Western blot images were captured by UVITEC Imaging Systems (Uvitec Ltd., UK). An inverted fluorescence microscope (Leica DMi8, Germany) was used to investigate subcellular localization, immunofluorescence staining, and monitor live cells proteins degradation over time. A confocal laser microscope (FV1000, Olympus, Japan) using 60x oil immersion was used for confocal microscopy imaging with 364-nm blue laser, 488-nm green laser, and 594-nm red laser. Biacore T200 was used for SPR binding assays. The Nanodrop one Spectrophotometer (Thermo Fischer) was used to characterize proteins. A BD Accuri® C6 flow cytometer was used for flow cytometry data acquisition. The PolygenDNA program and MOE software (v2019) were employed for all-atom characterization and molecular docking.
Data analysis	MestReNova (v12.0.3) was used for all chemical NMR analysis. Biacore T200 evaluation software was used for SPR binding assay analysis. Origin software (v2018) GraphPad Prism (v8) was used for data analysis and curve-fitting. ImageJ was used to analyze the cell cycle assay and quantify the blots. FlowJo (v10.0) was used for flow cytometry data analysis. The subcellular localization of proteomic analysis was preformed using WoLF-PSORT database; GO annotation is to annotate and analyze the identified proteins with eggnog-mapper software (v2.1.6). The software is based on the EggNOG database (v5.0.2, http://eggnog5.embl.de/#/app/home); Kyoto Encyclopedia of Genes and Genomes (KEGG) database (v5.0, http://www.kegg.jp/kegg/mapper.html) was used for KEGG pathway enrichment analysis. PPI network form STRING was visualized using the R package "networkD3" tool.

For manuscripts utilizing custom algorithms or software that are central to the research but not yet described in published literature, software must be made available to editors and reviewers. We strongly encourage code deposition in a community repository (e.g. GitHub). See the Nature Portfolio guidelines for submitting code & software for further information.

Data

Policy information about availability of data

All manuscripts must include a data availability statement. This statement should provide the following information, where applicable:

- Accession codes, unique identifiers, or web links for publicly available datasets
- A description of any restrictions on data availability
- For clinical datasets or third party data, please ensure that the statement adheres to our policy

All data that supported the findings of this study are available. Source data are provided with this paper. The crystal structures of CDK9 protein (pdb ID: 3BLH) and CRBN protein (pdb ID: 4CI3) were downloaded from the Protein Data Bank (PDB). The proteomics data used in this study are available in the ProteomeXchange database under accession code PXD042665 [https://www.ebi.ac.uk/pride/archive/projects/PXD042665].

Research involving human participants, their data, or biological material

Policy information about studies with human participants or human data. See also policy information about sex, gender (identity/presentation), and sexual orientation and race, ethnicity and racism.

Reporting on sex and gender	N/A
Reporting on race, ethnicity, or other socially relevant groupings	N/A
Population characteristics	N/A
Recruitment	N/A
Ethics oversight	N/A

Note that full information on the approval of the study protocol must also be provided in the manuscript.

Field-specific reporting

Please select the one below that is the best fit for your research. If you are not sure, read the appropriate sections before making your selection.

☒ Life sciences ☐ Behavioural & social sciences ☐ Ecological, evolutionary & environmental sciences

For a reference copy of the document with all sections, see nature.com/documents/nr-reporting-summary-flat.pdf

Life sciences study design

All studies must disclose on these points even when the disclosure is negative.

Sample size	Although there is no formal power calculation to define sample size, all cell experiments were conducted independently with n=3+, and the sample size was chosen to be consistent with the previous literature by utilizing similar assays. Specific replicate numbers are indicated in the text.
Data exclusions	There were no data exclusions.
Replication	Each condition was replicated at least three times independently to ensure the reliability of the results.
Randomization	All the samples used in this study were randomly allocated.
Blinding	No blinding was performed in chemical experiments and in vitro experiments in order to make comparisons between specific treatments.

Reporting for specific materials, systems and methods

We require information from authors about some types of materials, experimental systems and methods used in many studies. Here, indicate whether each material, system or method listed is relevant to your study. If you are not sure if a list item applies to your research, read the appropriate section before selecting a response.

Materials & experimental systems

n/a	Involved in the study
	☒ Antibodies
	☒ Eukaryotic cell lines
☒	Palaeontology and archaeology
☒	Animals and other organisms
☒	Clinical data
☒	Dual use research of concern
☒	Plants

Methods

n/a	Involved in the study
☒	ChIP-seq
	☒ Flow cytometry
☒	MRI-based neuroimaging

Antibodies

Antibodies used

1. Rabbit GAPDH polyclonal antibody (Proteintech Group, Rosemont, IL, USA, 10494-1-AP, 1:10000)
2. Rabbit CDK9 polyclonal antibody (Proteintech Group, Rosemont, IL, USA, 11705-1-AP, 1:1000)
3. Mouse CDK1/2 (AN21.2) monoclonal antibody (Santa Cruz Biotechnology, sc-53219, 1:250)
4. Mouse CDK6 antibody (Proteintech Group, Rosemont, IL, USA, 66278-1-Ig, 1:1000)
5. Rabbit ERG polyclonal antibody (Proteintech Group, Rosemont, IL, USA, 14356-1-AP, 1:1000)
6. Rabbit HPK1 polyclonal antibody (Proteintech Group, Rosemont, IL, USA, 23950-1-AP, 1:1000).
7. HRP-conjugated recombinant rabbit anti-mouse IgG kappa light chain (Proteintech Group, Rosemont, IL, USA, SA00001-19, 1:5000)
8. HRP-conjugated Affinity Pure goat anti-rabbit IgG (H+L) (Proteintech Group, Rosemont, IL, USA, SA00001-2, 1:10000)
9. Mouse CDK6 antibody (Proteintech Group, Rosemont, IL, USA, 66278-1-Ig, 1:100)
10. Rabbit CDK9 polyclonal antibody (Proteintech Group, Rosemont, IL, USA, 11705-1-AP, 1:100)
11. CoraLite594-conjugated donkey anti-mouse IgG(H+L) (Proteintech Group, Rosemont, IL, USA, SA00013-7, 1:100)
12. CoraLite488-conjugated donkey anti-rabbit IgG(H+L) (Proteintech Group, Rosemont, IL, USA, SA00013-6, 1:100)

Validation

All the antibodies were used for applications validated by antibody suppliers and the validating data was provided on their websites.
Tested Applications:
1. Rabbit GAPDH polyclonal antibody (Proteintech Group, Rosemont, IL, USA, 10494-1-AP): FC, IF, IHC, IP, WB, ELISA
2. Rabbit CDK9 polyclonal antibody (Proteintech Group, Rosemont, IL, USA, 11705-1-AP): FC, IF, IHC, WB, ELISA
3. Mouse CDK1/2 (AN21.2) monoclonal antibody (Santa Cruz Biotechnology, sc-53219): WB, IP, IF, IHC(P)
4. Mouse CDK6 antibody (Proteintech Group, Rosemont, IL, USA, 66278-1-Ig): IF, WB, ELISA
5. Rabbit ERG polyclonal antibody (Proteintech Group, Rosemont, IL, USA, 14356-1-AP): FC, IF, IHC, IP, WB, ELISA
6. Rabbit HPK1 polyclonal antibody (Proteintech Group, Rosemont, IL, USA, 23950-1-AP): IHC, IP, WB, ELISA
7. HRP-conjugated recombinant rabbit anti-mouse IgG kappa light chain (Proteintech Group, Rosemont, IL, USA, SA00001-19): ELISA, WB
8. HRP-conjugated Affinity Pure goat anti-rabbit IgG (H+L) (Proteintech Group, Rosemont, IL, USA, SA00001-2): ELISA, WB
9. Mouse CDK6 antibody (Proteintech Group, Rosemont, IL, USA, 66278-1-Ig): IF, WB, ELISA
10. Rabbit CDK9 polyclonal antibody (Proteintech Group, Rosemont, IL, USA, 11705-1-AP): FC, IF, IHC, WB, ELISA
11. CoraLite594-conjugated donkey anti-mouse IgG(H+L) (Proteintech Group, Rosemont, IL, USA, SA00013-7): IF, FC
12. CoraLite488-conjugated donkey anti-rabbit IgG(H+L) (Proteintech Group, Rosemont, IL, USA, SA00013-6): IF, FC

Eukaryotic cell lines

Policy information about cell lines and Sex and Gender in Research

Cell line source(s)

The acute myeloid leukemia cancer cell lines MV4-11, human hepatoma cells HepG2 and human prostate cancer cells PC3, and human embryonic kidney (HEK) 293T were purchased from American Type Culture Collection (ATCC, USA). Human T lymphocyte cell line Jurkat, Clone E6-1 was from Cell Bank/Stem Cell Bank, Chinese Academy of Sciences. The acute myeloid leukemia cancer cell lines MOLM13 was purchased from iCell Bioscience Inc, Shanghai.

Authentication

Cell lines have not been subjected to additional authentication.

Mycoplasma contamination

All cell lines were tested for mycoplasma and found negative.

Commonly misidentified lines
(See ICLAC register)

No commonly misidentified cells were used in this study.

Flow Cytometry

Plots

Confirm that:

☒ The axis labels state the marker and fluorochrome used (e.g. CD4-FITC).

☒ The axis scales are clearly visible. Include numbers along axes only for bottom left plot of group (a 'group' is an analysis of identical markers).

☒ All plots are contour plots with outliers or pseudocolor plots.

☒ A numerical value for number of cells or percentage (with statistics) is provided.

Methodology

Sample preparation	For real-time monition of CDK9 protein degradation in living cells, HEK293T cells overexpressed CDK9-eGFP fusion protein were incubated with indicated treatments for 12 h, and cells were washed with PBS three times, trypsinized, fixed, then transferred to EP tubes for flow cytometry analysis.<br>For the cell apoptosis experiment, MV4-11 cells were treated with different treatments for 6 h, then co-stained with annexin V/PI according to the instructions, and cell apoptosis was detected by flow cytometry.<br>For the cell cycle, MOLM13 cells were seeded in 6-well plates and treated with bis-DbTACs (final concentrations of 80, 200, 250, 500, and 1000 nM) or PBS (Control) for 12 h. After the treatment period, cells were harvested and fixed in ethanol. Fixed cells were then stained using the Cell Cycle Detection Kit following the manufacturer's protocol. The flow cytometry data were processed using ImageJ software.
Instrument	BD Accuri® C6 flow cytometer.
Software	Data analysis was performed using the FlowJo v10.0.
Cell population abundance	For real-time monition of CDK9 protein degradation in living cells, cell populations were composed of HEK293T cells and all cells were used for analysis. For sorting, cells expressing CDK9-eGFP were isolated from the cells by sorting, and the purity of cells after sorting was over 93%.<br>For the cell apoptosis, cell populations were composed of cancer cells and all cells were used for analysis. For sorting, cells were isolated from MV4-11 cells labeled with annexin V/PI, and the purity of cells after sorting was over 93%.<br>For the cell cycle, cell populations were composed of cancer cells and all cells were used for analysis. For sorting, cells were isolated from MOLM13 cells labeled with PI, and the purity of cells after sorting was over 93%.
Gating strategy	For real-time monition of CDK9 protein degradation in living cells, HEK293T cells with CDK9-eGFP were used to set the gating for analysis in Supplementary Fig. 26a.<br>For the cell apoptosis, Representative gating strategies are in Supplementary Fig. 26b.<br>For the cell cycle, Representative gating strategies are in Supplementary Fig. 26c. For analysis, a single cell population is first selected using a pulse area-pulse height plot gate. This gate is then applied to the scattering pattern to exclude obvious clear cell adhesion. Combine two set gates and apply them to cell cycle patterns.

☒ Tick this box to confirm that a figure exemplifying the gating strategy is provided in the Supplementary Information.
